# *Bifidobacterium dentium*-derived y-glutamylcysteine suppresses ER-mediated goblet cell stress and reduces TNBS-driven colonic inflammation

**DOI:** 10.1080/19490976.2021.1902717

**Published:** 2021-05-14

**Authors:** Melinda A. Engevik, Beatrice Herrmann, Wenly Ruan, Amy C. Engevik, Kristen A. Engevik, Faith Ihekweazu, Zhongcheng Shi, Berkley Luck, Alexandra L. Chang-Graham, Magdalena Esparza, Susan Venable, Thomas D. Horvath, Sigmund J. Haidacher, Kathleen M. Hoch, Anthony M. Haag, Deborah A. Schady, Joseph M. Hyser, Jennifer K. Spinler, James Versalovic

**Affiliations:** a Department of Pathology & Immunology, Baylor College of Medicine, Houston, Texas, USA; b Department of Pathology, Texas Children’s Hospital, Houston, Texas, USA; c Department of Pediatrics, Baylor College of Medicine, Houston, Texas, USA; d Section of Gastroenterology, Hepatology, and Nutrition, Texas Children’s Hospital, Houston, Texas, USA; e Department of Surgery, Vanderbilt University Medical Center, Nashville TN, USA; f Department of Molecular Virology & Microbiology, Baylor College of Medicine, Houston, TX, USA; g Alkek Center for Metagenomics and Microbiome Research, Baylor College of Medicine, Houston, TX, USA

**Keywords:** MUC2, goblet cells, *bifidobacteria*, ER-stress, organoids, IL-10, colitis, IBD

## Abstract

Endoplasmic reticulum (ER) stress compromises the secretion of MUC2 from goblet cells and has been linked with inflammatory bowel disease (IBD). Although *Bifidobacterium* can beneficially modulate mucin production, little work has been done investigating the effects of *Bifidobacterium* on goblet cell ER stress. We hypothesized that secreted factors from *Bifidobacterium dentium* downregulate ER stress genes and modulates the unfolded protein response (UPR) to promote MUC2 secretion. We identified by mass spectrometry that *B. dentium* secretes the antioxidant γ-glutamylcysteine, which we speculate dampens ER stress-mediated ROS and minimizes ER stress phenotypes. *B. dentium* cell-free supernatant and γ-glutamylcysteine were taken up by human colonic T84 cells, increased glutathione levels, and reduced ROS generated by the ER-stressors thapsigargin and tunicamycin. Moreover, *B. dentium* supernatant and γ-glutamylcysteine were able to suppress NF-kB activation and IL-8 secretion. We found that *B. dentium* supernatant, γ-glutamylcysteine, and the positive control IL-10 attenuated the induction of UPR genes GRP78, CHOP, and sXBP1. To examine ER stress *in vivo*, we first examined mono-association of *B. dentium* in germ-free mice which increased MUC2 and IL-10 levels compared to germ-free controls. However, no changes were observed in ER stress-related genes, indicating that *B. dentium* can promote mucus secretion without inducing ER stress. In a TNBS-mediated ER stress model, we observed increased levels of UPR genes and pro-inflammatory cytokines in TNBS treated mice, which were reduced with addition of live *B. dentium* or γ-glutamylcysteine. We also observed increased colonic and serum levels of IL-10 in *B. dentium-* and γ-glutamylcysteine-treated mice compared to vehicle control. Immunostaining revealed retention of goblet cells and mucus secretion in both *B. dentium-* and γ-glutamylcysteine-treated animals. Collectively, these data demonstrate positive modulation of the UPR and MUC2 production by *B. dentium-*secreted compounds.

## Graphical abstract

**Figure:**
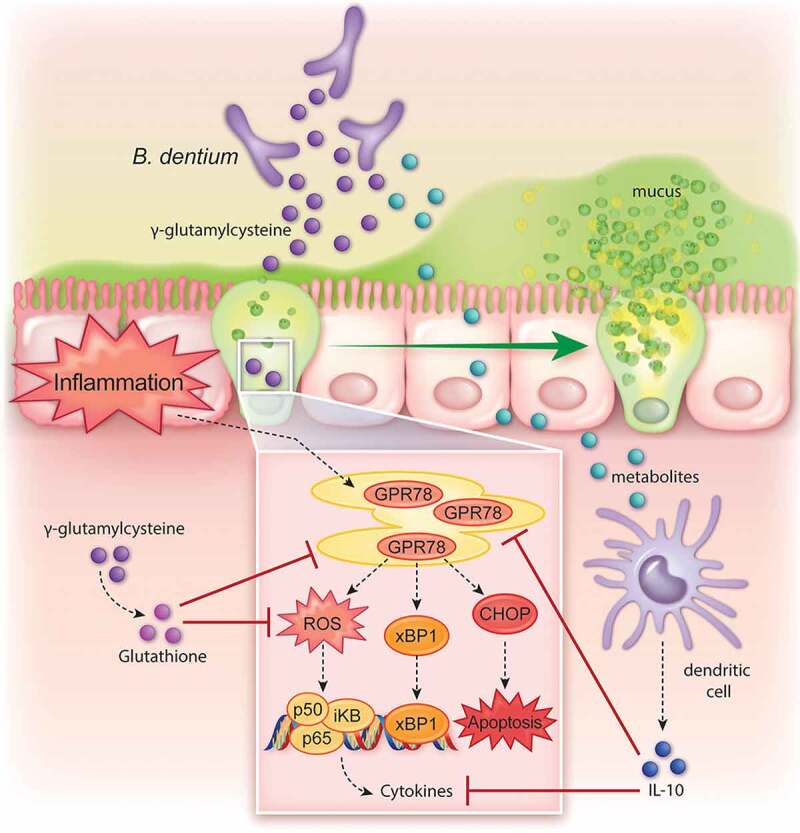

## Introduction

The gastrointestinal epithelium functions as a barrier to prevent undesirable luminal antigens or irritants from entering the body.^1^ The intestinal barrier is maintained by both the maintenance of intact epithelial cells and by the protective mucus layer that overlays the epithelium. Intestinal mucus is synthesized and secreted from goblet cells.^2,3^ Mucus synthesis starts with dimerization of mucin MUC2 proteins in the endoplasmic reticulum (ER), followed by O-glycosylation in the Golgi. After further oligomerization, mature mucins are stored as granules until they are released from intestinal goblet cells.^4^ Since mucin synthesis requires precise continuous folding in the ER, goblet cells are particularly sensitive to ER stress.^4,5^ ER stress occurs when misfolded proteins accumulate, and this stress induces signaling pathways that initiate the unfolded protein response (UPR).^6,7^ UPR is initiated by the heat shock protein family chaperone GRP78, which then activates distinct signal transducers.^5^ ER stress can also generate reactive oxygen species (ROS) and activate NF-kB.^8,9^ The balance of ROS levels in the ER is critical for homeostasis as excessive accumulation of ROS leads to further accumulation of misfolded proteins, thereby creating a cycle of ER stress.^10,11^ Excessive or chronic ER stress and oxidative stress in goblet cells reduces MUC2 production, depletes the mucus barrier, and induces cell injury and inflammation.

ER and oxidative stress have been implicated as important contributors to chronic inflammatory diseases, including inflammatory bowel disease (IBD).^7,12–20^ Multiple experimental mouse models have reproduced the ER stress, oxidative stress, and decreased mucin production observed in patients, which demonstrate a causative role for ER stress in intestinal inflammation.^4,21–32^ Compounds commonly used in animal models of colitis, including 2,4,6-Trinitrobenzenesulfonic acid (TNBS) and dextran-sodium-sulfate (DSS), activate ER stress.^33–36^ Furthermore, ER-stress inhibitors ameliorate colitis in mice, which supports a role for ER stress modulation in intestinal inflammation.^24^ Additionally, inhibitors of ROS, which contribute to the pathogenesis of ER stress, also ameliorate both ER stress and colitis.^35,37–39^

The antioxidant glutathione plays a pivotal role in regulating the ER protein folding process.^40–45^ Cells generate glutathione through a two-step process. First, cells combine glutamate and cysteine to create γ-glutamylcysteine. Then glycine is added to y-glutamylcysteine to generate glutathione. Reduced levels of γ-glutamylcysteine and glutathione have been observed in IBD patients,^46–50^ which could contribute to the observed levels of ROS and ER stress. Since the antioxidant capacity of IBD patients is compromised, many researchers have speculated that antioxidant therapies may benefit IBD patients.^51^ Although administration of glutathione is safe, research suggests that glutathione is poorly absorbed by the oral route.^52–55^ In contrast, oral γ-glutamylcysteine is readily absorbed by the PEPT1 and PEPT2 transporters, increases glutathione levels, and is safe for use in patients.^56–65^ Unfortunately, the commercial production of pure γ-glutamylcysteine is relatively cost-prohibitive. Alternatively, intestinal microbes also produce γ-glutamylcysteine; therefore, microbial γ-glutamylcysteine could be a source for upregulating host glutathione levels. *Bifidobacteria* is of particular interest as select strains have been shown to alleviate ER stress.^66^ *B. dentium* ATCC 27678 harbors the GshA gene required for making γ-glutamylcysteine. Thus, we hypothesized that *B. dentium* secreted products, particularly the antioxidant γ-glutamylcysteine, could suppress TNBS- or thapsigargin-driven ER and oxidative stress, bolster the mucus layer, and reduce intestinal inflammation.

## Methods

### Bacterial Culture

*Bifidobacterium dentium* ATCC 27678 (ATCC, American Type Culture Collection), a human fecal isolate, was grown in an anaerobic workstation (Anaerobe Systems AS-580) with a mixture of 5% CO_2_, 5% H_2_, and 90% N_2._ *B. dentium* was grown in de Man, Rogosa, and Sharpe (MRS) medium (Difco) from single colonies at 37°C overnight anaerobically. *B. dentium* was subcultured into a fully defined media, termed LDM4, at an optical density (OD_600nm_) = 0.1 as previously described.^67^ LDM4 cultures were grown anaerobically for 24 hr at 37°C. After incubation, cultures were centrifuged at 5,000 x g for 5 min. The supernatant was adjusted to a pH of 7 and sterile filtered through a 0.2 µm-pore PVDF-membrane (Polyvinylidene Fluoride, Millipore). This supernatant is termed “conditioned media.” For animal experiments, *B. dentium* was grown overnight anaerobically in MRS and centrifuged at 5,000 x g for 5 min. Bacteria were then washed 2x with sterile anaerobic PBS and adjusted to 10^9^ CFU mL^−1^. These bacteria were used for oral gavage. Bacterial viability was confirmed for each gavage session by serial plating *B. dentium* on MRS agar to calculate CFUs.

### Mass Spectrometric Analysis of γ-glutamylcysteine

The liquid chromatography-tandem mass spectrometry (LC-MS/MS) system was comprised of a Shimadzu Nexera X2 MP Ultrahigh-Performance Liquid Chromatography (UHPLC) system (Kyoto, Japan) coupled to a Sciex 6500 QTrap hybrid triple-quadrupole/linear ion trap MS system from Danaher (Washington, DC, USA). Operational control of the LC-MS/MS was performed with Analyst® (Ver. 1.6.2), and quantitative analysis was performed using MultiQuant™ (Ver. 3.0.1). The targeted LC-MS/MS-based metabolomics methods used for the quantitative analysis of the γ-Glu-Cys content of the LDM4 medium are described in their entirety in the Supplemental Materials Section.

### Tissue Culture

#### Culturing conditions

Human colon T84 cells (ATCC CCL-248) were obtained from ATCC and grown in Gibco Dulbecco’s Modified Eagle Medium (ThermoFisher) supplemented with 10% fetal bovine serum (FBS) in a humidified atmosphere at 37°C, 5% CO_2_ (see supplemental methods for additional details). T84 cells were grown to confluence on 24-well tissue culture treated plates and 1 µg/mL ER stressor thapsigargin (Tocris #1138), 10 µg/mL ER stressor tunicamycin (Sigma #T7765-1 MG), or 0.1 µg/mL IL-1β in the presence or absence of various concentrations of *B. dentium* LDM4 conditioned media, 2 mM γ-glutamylcysteine (Bachem # 4028244.025), or 50 ng/mL IL-10 (Peprotech #200-10) in DMEM without glucose and without FBS for 6 hr. Following incubation, cells were incubated with TRIZOL for RNA extraction. For western blot analysis, cells were seeded at 5 × 10^4^ cells/cm^2^ in 12-well tissue culture treated plates (Corning) until the cells reached confluence. Once cells reached confluency, T84 cells were serum starved by incubation overnight in DMEM without glucose and without FBS at 37°C, 5% CO_2_. Cells were then treated with 1 µg/mL thapsigargin with or without 50% *B. dentium* LDM4 conditioned media or γ-glutamylcysteine in DMEM without glucose and without FBS for 8 hr. After incubation, cells were lysed in lysis buffer and stored at −80°C until processing. Cell viability was examined by propidium iodide staining (see supplemental methods).

#### ROS and Glutathione Analysis

To examine ROS, T84 cells were pretreated with 5 μM 2′,7′-Dichlorofluorescin diacetate (H_2_DCFDA; Sigma Aldrich Cat# D6883) for 1 hr at 37°C, 5% CO_2_. Cells were then washed gently 2x with PBS and treated with 10 µg/mL ER stressor tunicamycin, or 2 mM H_2_0_2_ in the presence or absence of various concentrations of *B. dentium* LDM4 conditioned media, 2 mM γ-glutamylcysteine, or IL-10 in DMEM. Cells were incubated with treatment conditions for 3 hr, washed 3x with PBS, and then H_2_DCFDA fluorescence was examined in cells in PBS on a Synergy H1 plate reader at excitation 485 nm/emission 520 nm. ThiolTracker Violet (ThermoFisher #T10095), an intracellular thiol probe used to detect glutathione levels. T84 cells were incubated with *B. dentium* LDM4 conditioned media, 2 mM γ-glutamylcysteine, or IL-10 in DMEM for 3 hr at 37°C, 5% CO_2._ Cells were then washed and incubated with 20 μM ThiolTracker Violet in PBS for 30 min at 37°C, 5% CO_2._ After incubation, cells were washed and fluorescence was examined on a Synergy H1 plate reader at excitation 404 nm/emission 526 nm. γ-glutamylcysteine uptake was examined using fluorescein (see supplemental methods).

#### NF-kB Activation and IL-8 Analysis

To examine NF-kB activation, T84 cells at 80% confluence were transiently transduced with an NF-kB secreted luciferase reporter (Clontech) in Opti-MEM (ThermoFisher) using the XtremeGene HP DNA transfection reagent (Roche).^68^ The final concentration of 0.6 μL XtremeGene HP:0.3 μg DNA per well. Cells were then incubated for 48 hours at 37°C, 5% CO2. Following transfection, cells were treated with 1 µg/mL thapsigargin with or without 50% LDM4 un-inoculated media, 50% *B. dentium* LDM4 conditioned media or γ-glutamylcysteine in DMEM without glucose and without FBS overnight. Supernatant was examined for luciferase activity using a Lonza Lucetta tube luminometer with a 2 second delay and a 10 second measurement time. To examine IL-8 production, T84 cells were seeded into 96-well plates (10,000 cells/well) overnight and the following day, cells were serum starved for 3 hr in DMEM without glucose and FBS. Then cells were treated with 1 µg/mL thapsigargin or 0.1 µg/mL IL-1β in the presence or absence of various concentrations of *B. dentium* LDM4 conditioned media or γ-glutamylcysteine. Cells were incubated overnight (16 hr) and supernatants were examined for IL-8 production by IL-8/CXCL8 DuoSet ELISA (R&D, #DY208-05).

### Mouse Bone Marrow-Derived Dendritic Cell Culture

Mouse bone marrow dendritic cells were isolated as previously described.^69^ Briefly, bone marrow was flushed from the femur and tibia of 8 week old male Swiss Webster mice, treated with red blood lysis buffer and 10^5^ mL^−1^ bone marrow cells were seeded into 10 cm Petri dishes in 10 mL RPMI-1640 with 10% (v/v) heat-inactivated FBS and 100 ng/mL murine GM-CSF (peprotech #315-03) and IL-4 (peprotech #214-14). Cells were incubated for 7 days at 37°C, 5% CO2 and media were changed on day 3. On day 6, cells were trypsinized, seeded in new dishes at 2 × 10^5^ cells/mL and incubated overnight. On day 7, dendritic cells were treated with 100 ng/mL LPS, un-inoculated LDM4 or *B. dentium* LDM4 conditioned medium and incubated overnight. The following day, the supernatant was removed and examined by IL-10 ELISA (ThermoFisher #88-7105-22).

### Mouse Colonic Organoid Culture

Mouse colonic organoids were generated as previously described.^70^ Briefly, the colon was excised from 8-week -old male Swiss Webster mice and washed thoroughly in ice-cold Ca^2+^/Mg^2+^-free DPBS. Tissue was incubated in 3 mM EDTA, DTT, and sucrose for 30 min at 4°C. Crypts were collected in chelation buffer, centrifuged at 300 *x g* for 10 min, and embedded in Matrigel (BD Biosciences). After Matrigel polymerization, Matrigel domes were covered with complete media with growth factors (CMGF+) containing 10 µM Y-27632 rock inhibitor.^71^ Colonic organoids were used in experiments after two passages to ensure cellular debris was removed. For differentiation, colonic organoids were grown for 48 hr in CMGF+, then the medium was changed to differentiation media. Delivery of bacterial conditioned media to the luminal membrane of colonic organoids was achieved by microinjection of 17.6 nL of solution (media control, uninoculated LDM4, LPS or *B. dentium* LDM4 conditioned media) using a Nanoject microinjector (Drummond Scientific Company) as previously described.^72^ Colonic organoids were incubated overnight and supernatant was analyzed using an IL-10 ELISA (ThermoFisher #88-7105-22).

### Animal Models

All animal experimental procedures were approved by the Institutional Animal Care and Use Committee (IACUC) at Baylor College of Medicine, Houston, TX. For gnotobiotic experiments, animals were housed in filter-top cages in sterile isolators at the Baylor College of Medicine germ-free facility. Swiss Webster germ-free mice were gavaged with sterile MRS media (Germ-Free controls) or were gavaged with 3.2 × 10^8^ CFU mL^−1^ *B. dentium* ATCC 27678 grown in MRS (*B. dentium* mono-associated). Both groups contained equal numbers of male and female mice to exclude gender bias (n = 5 males/5 females per treatment group). To ensure colonization, mice received oral gavage treatments once every other day for one week and a final gavage a week later as previously described.^67^ Colonization was confirmed by plating fecal samples on MRS and Blood Agar (Hardy Diagnostics). To confirm the absence of other bacteria, agar plates were incubated anaerobically and aerobically at 37°C for 48 hr.

For TNBS experiments, BALB/c mice (8–12 weeks old) were purchased from Taconic and housed in the Baylor College of Medicine animal facility (Feigin Tower). Mice were pretreated by oral gavage with *B. dentium* 10^9^ CFU mL^−1^ or 1 mg/kg y-glutamylcysteine (Bachem). After 1 week of pretreatment, mice were anesthetized by isoflurane inhalation and 5% (wt/vol) 2,4,6-Trinitrobenzenesulfonic acid (TNBS) in ethanol was rectally administered. To ensure TNBS retention, mice were maintained in a vertical position for 2 min. Following TNBS administration, mice received daily oral gavage of either microbial or y-glutamylcysteine treatment until euthanasia (3–5 days). Histological scores of colitis were assessed by a Texas Children’s Hospital pathologist. Staining was performed on paraffin embedded colon sections (see supplemental methods). Colon tissue was also collected in TRIZOL and used to isolate RNA (see supplemental methods). Serum cytokines were analyzed using a Cytokine Magnetic bead panel (Millipore, cat. #MCYTOMAG) with a MagPix instrument (see supplemental methods).

### Statistics

Data are presented as mean ± standard deviation. Comparisons between groups were made with Student’s t-test, One-way or Two-way Analysis of Variance (ANOVA), using the Holm-Sidak post-hoc test to determine significance between pairwise comparisons. Graphs and statistics were generated using GraphPad (GraphPad Software, Inc. La Jolla, CA). A *p < .05 value was considered significant while *n* is the number of experiments performed.

## Results

*B. dentium secretes γ-glutamylcysteine which promotes epithelial glutathione production and diminishes ROS and NF-kB activation*

**Figure 1.**
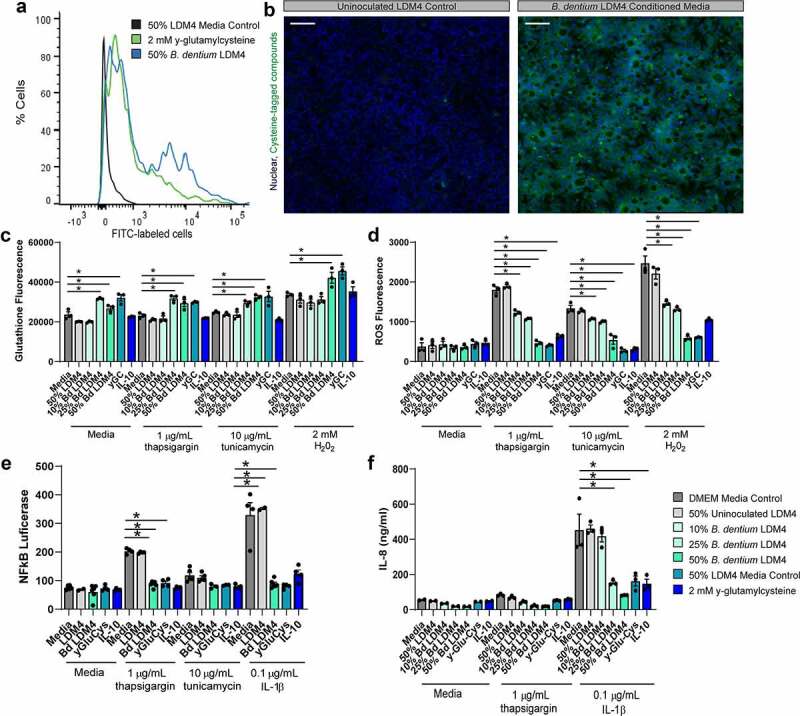
***B. dentium* γ-glutamylcysteine enter host cells, upregulate glutathione and reduce ROS, NF-kB, and cytokine synthesis. a**. Fluorescein-5-Maleimide was used to fluorescently tag cysteine residues in y-glutamylcysteine, *B. dentium* conditioned LDM4 media, or uninoculated LDM4 media. Representative histograms from flow cytometry analysis of T84 cells after exposure to cysteine-tagged y-glutamylcysteine, *B. dentium*-conditioned LDM4 media, or uninoculated LDM4 media (control) (n = 3/experiment). **b**. Representative images of T84 cells following incubation with Fluorescein-5-Maleimide-tagged *B. dentium* conditioned LDM4 (which fluorescently labels cysteine residues), counterstained with nuclear dye Hoechst (scale bar = 50 µm). **c**. Measurement of glutathione levels in T84 cells after 3 hr using a Thiol-tracker, as measured on a fluorescence plate reader (ex/em: 405/528) (n = 3/experiment). **d**. Measurement of ROS levels in T84 cells after 3 hr in cells stained with H_2_DCFDA, as measured on a fluorescent plate reader (ex/em: 485/528) (n = 3/experiment). **e**. Secreted NF-kB luciferase quantified from T84 monolayers treated for 16 hr (n = 4/experiment). **f**. IL-8 levels of T84 cells after 16 hr incubation with treatment as measured by ELISA(n = 3/experiment). All data is expressed as mean ± st dev and all experiments were repeated 3–4 independent times. *p < .05, Multi-Way ANOVA

γ-glutamylcysteine, the precursor to glutathione, is a modulator of both oxidative and ER stress. To determine the ability of *B. dentium* to produce γ-glutamylcysteine, we grew the bacteria in a fully defined medium termed LDM4 for 16 hr and assessed the concentration of γ-glutamylcysteine in the supernatant by mass spectrometry (MS/MS). *B. dentium* secreted high levels of γ-glutamylcysteine (2.2 ± 0.7 µg/mL) in LDM4. No levels of microbial glutathione were detected. In the intestine, γ-glutamylcysteine can be taken up by PEPT1 and PEPT2 transporters where it can feed into the host glutathione pathway. To model the colon, we selected the mucin-producing colonic cell line T84, which expresses the γ-glutamylcysteine transporter PEPT1, secretes mucus, and has been previously used to examine ER stress.^73–76^ To assess whether *B. dentium* secreted γ-glutamylcysteine could be incorporated by the host, we fluorescently labeled all cysteine-containing compounds, including γ-glutamylcysteine, in *B. dentium* conditioned LDM4 with fluorescein-5-maleimide and examined intracellular localization in T84 cells by flow cytometry and microscopy (Figure 1a,b). As a control, we also labeled purified γ-glutamylcysteine with fluorescein-5-maleimide. Consistent with the high levels of γ-glutamylcysteine observed in *B. dentium*-conditioned LDM4, we found high expression of cysteine-labeled compounds in T84 cells. In contrast to unstained T84 cells (3.6 ± 0.03%) and 50% inoculated fluorescently labeled-LDM4 controls (9.2 ± 1.9%), *B. dentium* fluorescently labeled LDM4-conditioned media was present in 89.1 ± 1.91% of cells by flow cytometry (Figure 1a). Moreover, purified γ-glutamylcysteine was found in 76.3 ± 1.63% of cells. Fluorescence microscopy confirmed the presence of fluorescently labeled *B. dentium* supernatant in T84 cells (Figure 1b). These data indicate that microbial γ-glutamylcysteine can enter the intestinal epithelium.

To confirm that microbial γ-glutamylcysteine could regulate host glutathione, we added purified γ-glutamylcysteine and *B. dentium* conditioned LDM4 containing γ-glutamylcysteine to T84 cells and measured glutathione production using a fluorescent thiol-tracker (Figure 1c). We observed elevated levels of glutathione in response to *B. dentium* and γ-glutamylcysteine treatment, indicating that microbial-derived γ-glutamylcysteine is capable of elevating host glutathione levels. As a control, we also included IL-10, which has been shown to suppress goblet cell ER stress and ROS.^4,9,77,78^ Interestingly, we did not observe any change in glutathione levels in IL-10 treated cells compared to their respective media controls. Glutathione is known to minimize ROS, a byproduct of ER stress and thereby suppress NF-kB activation.^8,9,45^ To address the role of microbial γ-glutamylcysteine in suppressing ROS, we fluorescently labeled T84 cells with H_2_DCFDA and examined ROS fluorescence after treatment (Figure 1d). As expected, γ-glutamylcysteine and IL-10 suppressed ROS generated by ER stress (thapsigargin and tunicamycin) as well as oxidative stress (H_2_0_2_). *B. dentium* conditioned LDM4 and γ-glutamylcysteine suppressed all forms of ROS, indicating that microbial compounds can promote host glutathione and suppress ROS. Finally, we examined NF-kB activation using T84 cells transiently transfected with an NF-kB secreted luciferase reporter (Figure 1e). In this assay, we also observed decreased levels of NF-kB in response to ER stress (thapsigargin) and pro-inflammatory cytokines (IL-1β) in our γ-glutamylcysteine, *B. dentium* cell-free supernatant and IL-10 treated cells. Our NF-kB activation by IL-1β was consistent with levels of IL-8, a downstream target (Figure 1f). We found that *B. dentium* conditioned LDM4, γ-glutamylcysteine, and IL-10 diminished IL-1β-induced IL-8 production. These data provide strong evidence that *B. dentium* secreted products, such as γ-glutamylcysteine, could suppress the ER stress phenotype.

*B dentium and y-glutamylcysteine suppress thapsigargin and tunicamycin-induced ER stress in mucin-producing cell lines*

**Figure 2.**
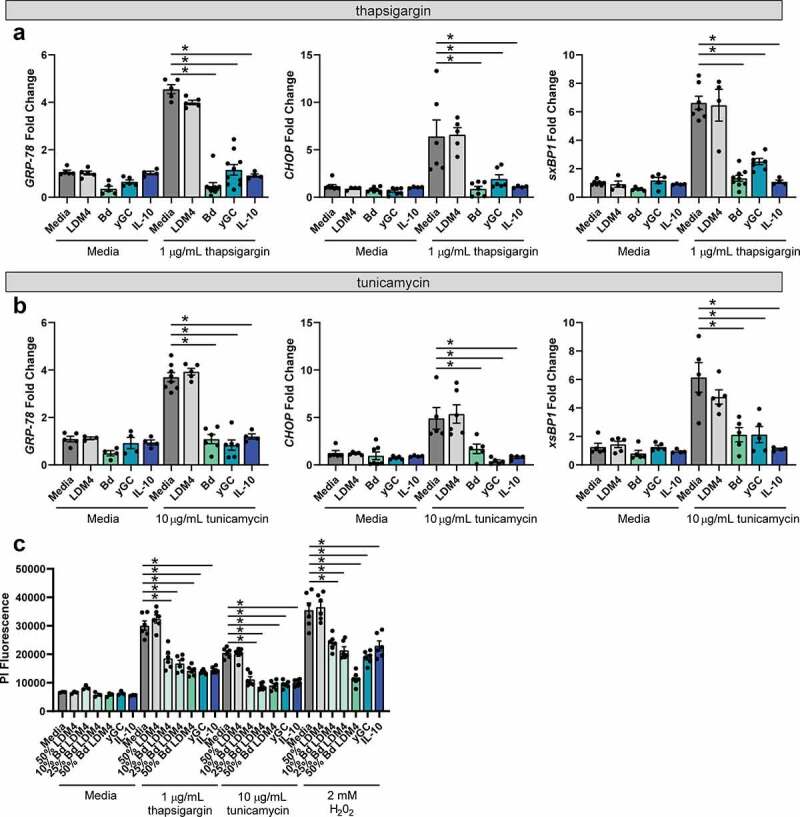
**ER stress can be suppressed by *B. dentium*, γ-glutamylcysteine, and IL-10. a**. qPCR analysis of T84 monolayers after 6 hr incubation with or without the ER-stressor thapsigargin. Cells were treated with either media, 50% un-inoculated LDM4 (LDM4), 50% *B. dentium* LDM4 (Bd), 2 mM γ-glutamylcysteine (yGC), or 100 ng/mL IL-10 (IL-10) (n = 6/experiment). **b**. qPCR analysis of T84 monolayers after 6 hr incubation with or without the ER-stressor tunicamycin (n = 6/experiment). **c**. Propidium iodide staining of T84 cells after 48 hr incubation with ER stressors (thapsigargin or tunicamycin) or oxidative stressor hydrogen peroxide (H_2_0_2_) (n = 6/experiment). *p < .05, Multi-Way ANOVA

Next, we sought to determine if *B. dentium* conditioned LDM4 could dampen ER stress signaling components. GRP-78 is the major regulator of ER stress and its activation contributes to the initiation and regulation of inflammatory processes and apoptosis.^79,80^ We first examined ER stress signals GRP-78, CHOP, and xsBP1 by qPCR in T84 cells (Figure 2a,b). We observed elevated levels of GRP-78, CHOP, and xsBP1 in response to ER stressors thapsigargin and to a lesser degree tunicamycin. However, treatment with 50% *B. dentium* LDM4 conditioned medium, γ-glutamylcysteine, and IL-10 significantly suppressed the expression of all ER stress proteins in the presence of both thapsigargin and tunicamycin. Chronic ER stress promotes apoptosis, so we also examined cell death using propidium iodide after 48 hr of incubation (Figure 2c). Significant propidium iodide staining, and thus cell death, was observed in thapsigargin, tunicamycin, and H_2_0_2_ treated cells. Similar propidium iodide staining was observed in uninoculated LDM4 bacterial media controls. In contrast, significantly less cell death occurred in *B. dentium*-conditioned LDM4, γ-glutamylcysteine, and IL-10 treated wells. These data indicate that *B. dentium* secreted products, including γ-glutamylcysteine, can suppress ER stress and apoptosis in mucin-producing cells.

*Mono-association of mice with B. dentium stimulates IL-10 and MUC2 production*

**Figure 3.**
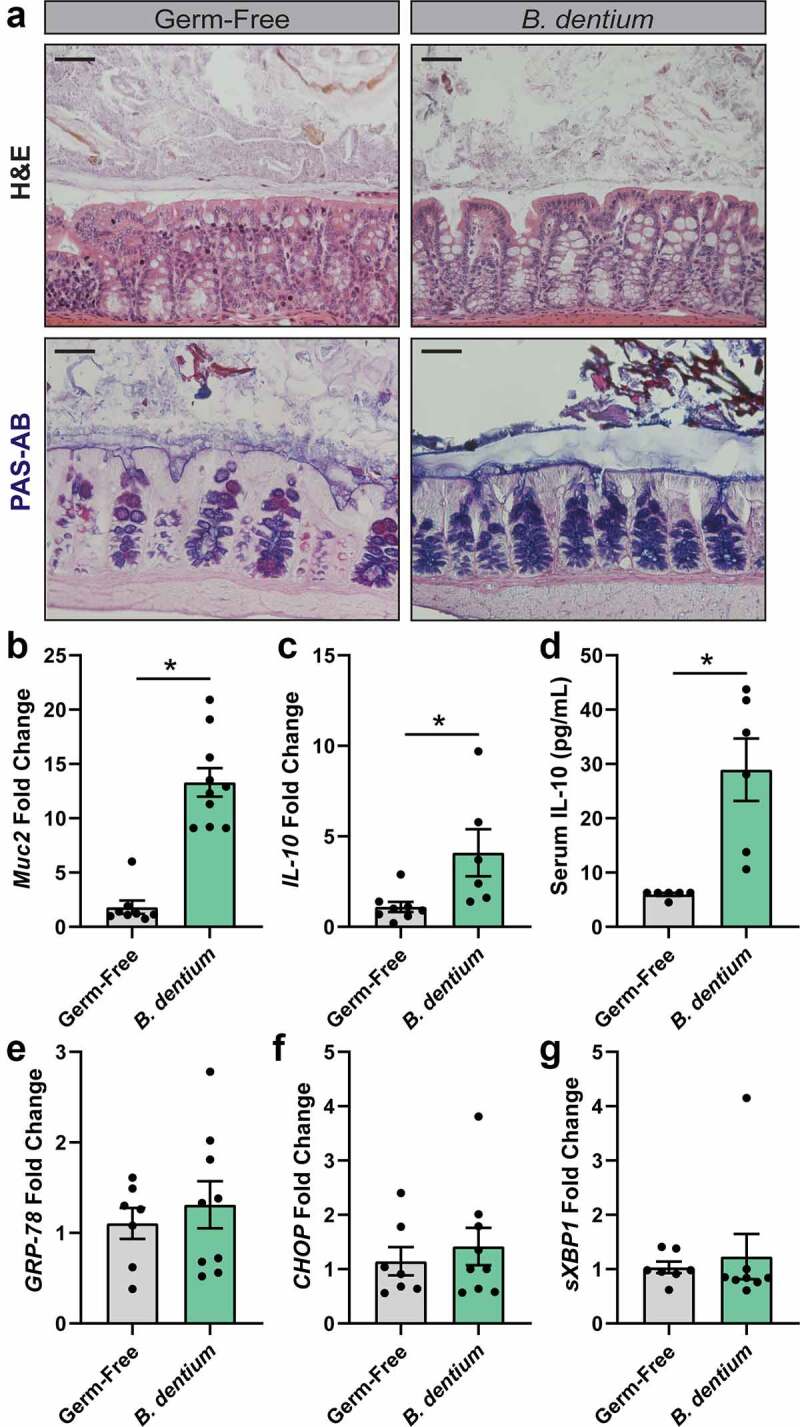
***B. dentium* promotes colon MUC2 and IL-10 secretion in gnotobiotic mice. a**. Representative images of H&E and Periodic Acid Schiff-Alcian Blue (PAS-AB) stains of germ-free and *B. dentium* mono-associated colon (scale bar = 50 µm). **b**. Colonic mRNA expression of *Muc2*. **c**. Colonic mRNA expression of *IL-10*. **d**. Serum levels of IL-10 by ELISA. **e-g**. Colonic mRNA expression of ER-stress related genes (e) *GRP-78*, (f) *CHOP* and (g) *sXBP1*. All analyses were performed in germ free (n = 10) and *B. dentium* mono-associated mice (n = 10). *p < .05, students t-test

Given the dramatic suppression of ER stress by IL-10 and *B. dentium*-conditioned LDM4, we next sought to determine if *B. dentium* colonization promoted mucus-production and IL-10 secretion *in vivo*. We mono-associated germ-free mice by oral gavage with live *B. dentium* and examined the colonic architecture and mucus layer by H&E and PAS-AB staining (Figure 3a). We observed normal crypt architecture by H&E in *B. dentium* mono-associated mice, with increased numbers of goblet cells compared to germ-free controls. Periodic Acid Schiff-Alcian Blue (PAS-AB) mucus staining confirmed that *B. dentium* colonization increased mucin-positive goblet cells. This observation was consistent with increased MUC2 mRNA levels in *B. dentium* mono-associated mice compared to germ-free counterparts (Figure 3b). We also examined whole colon IL-10 production by qPCR (Figure 3c). We observed elevated IL-10 mRNA and serum levels in *B. dentium* mono-associated mice compared to germ-free controls (Figure 3c,d). Importantly, we did not observe any changes in the expression of ER stress genes (GRP-78, CHOP, or xsBP1) in *B. dentium*-colonized mice, suggesting that *B. dentium* colonization promotes mucus production without stimulating goblet cell ER stress.

**Figure 4.**
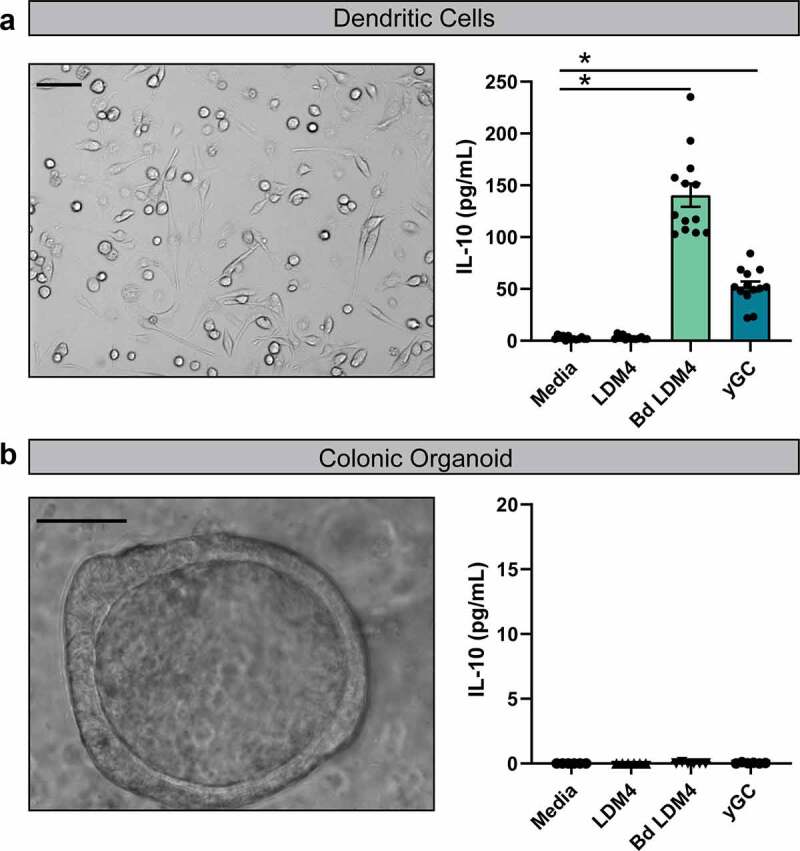
**Dendritic cells secrete IL-10 in response to *B. dentium*-conditioned media.a** Representative phase-contrast image of mouse bone-marrow derived dendritic cells and IL-10 measurements of dendritic cell supernatants by ELISA. Dendritic cells (100x, scale bar = 100 μm) were incubated with either media, 25% un-inoculated LDM4 media, 25% *B. dentium* conditioned LDM4 media or 2 mM γ-glutamylcysteine for 16 hr. **b**. Representative phase-contrast image of colonic organoid generated from germ-free mice and IL-10 measurements of organoid supernatant by ELISA. Colonic organoids (400x, scale bar = 50 μm) were incubated with either media, 25% uninoculated LDM4 media, 25% *B. dentium-*conditioned LDM4 media or 2 mM γ-glutamylcysteine for 16 hr. n = 3/experiments, repeated 2 independent times. *p < .05, One-Way ANOVA

IL-10 is commonly produced by dendritic cells and can be produced in response to bacterial stimulation.^81,82^ To determine if *B. dentium* secreted factors could stimulate IL-10 from immune cells, we generated bone marrow-derived mouse dendritic cells. Addition of uninoculated LDM4 had no effect on IL-10 levels as measured by ELISA (Figure 4a). However, addition of *B. dentium* LDM4 conditioned media and γ-glutamylcysteine both stimulated IL-10 production. To confirm that the epithelium was not responsible for IL-10 synthesis, colonic organoids were generated from germ-free mice and treated with uninoculated LDM4, *B. dentium*-conditioned LDM4, or γ-glutamylcysteine (Figure 4b). The epithelial cells in the organoids were unable to produce IL-10, indicating that *B. dentium*-secreted products can promote IL-10 from immune cells such as dendritic cells.

B. dentium and γ-glutamylcysteine elevate IL-10 and protect against TNBS colitis

**Figure 5.**
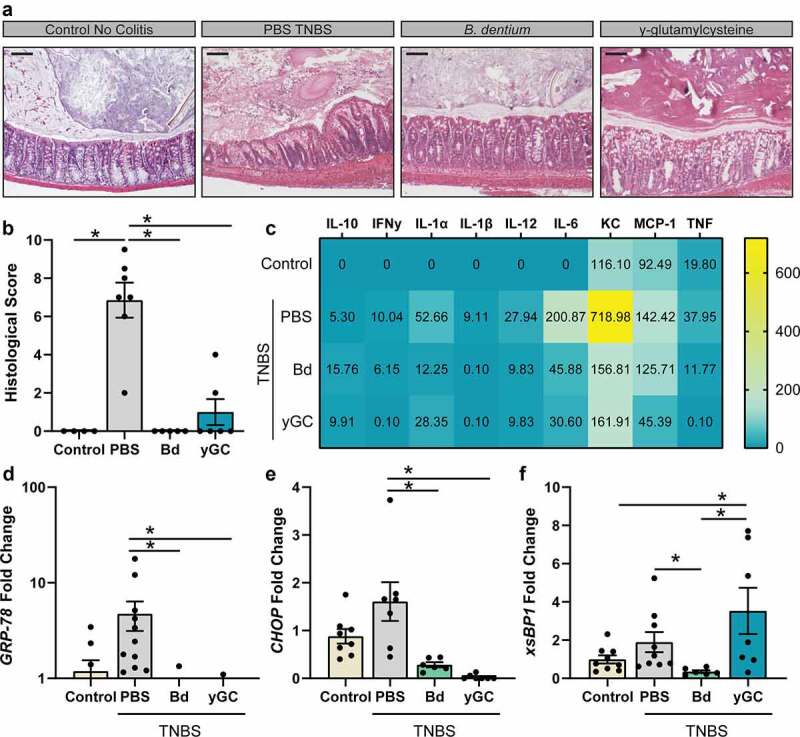
***B. dentium* and γ-glutamylcysteine suppress TNBS colitis. a**. Representative images of H&E stains of untreated control animals and TNBS treated animals receiving PBS vehicle, live *B. dentium* or γ-glutamylcysteine (scale bar = 100 µm). **b**. Histological scores of mice **c**. Serum cytokines heatmap as measured by Illumina Magpix. **d-f**. Colonic mRNA expression of ER-stress related genes (d) *GRP-78*, (**D**) *CHOP* and (f) *sXBP1*. All analyses were performed in untreated (n = 8), TNBS-PBS (n = 10), TNBS-*B. dentium*-treated (n = 10) or TNBS- γ-glutamylcysteine-treated mice (n = 8). *p < .05, One-Way ANOVA

Colitis-inducing compounds, including TNBS, are known to activate ER stress.^33–36^ We therefore investigated whether *B. dentium* and γ-glutamylcysteine could downregulate the molecular features of ER stress and minimize experimental colitis. We induced colitis in mice by rectal administration of TNBS in ethanol, which causes severe colitis as assessed by histological scoring. As anticipated, we observed extensive microscopic damage to colonic architecture in PBS-vehicle control mice with TNBS compared with untreated mice (Figure 5a). We observed immune infiltration, transmural inflammation with thickening of the muscularis, and loss of crypts and goblet cells in the colons of PBS-treated TNBS mice; all hallmarks of disease activity. In contrast, *B. dentium*- and γ-glutamylcysteine-treated TNBS mice exhibited significant improvement in colonic histopathology compared with PBS-treated TNBS mice, which is reflected in the histological scores (Figure 5b). Serum analysis by Magpix revealed elevated anti-inflammatory IL-10 in *B. dentium-* and γ-glutamylcysteine-treated mice with TNBS compared with PBS-vehicle treated TNBS and untreated mice (Figure 5c). Additionally, pro-inflammatory cytokines (IFNγ, IL-1α, IL-1β, IL-12, IL6, KC and TNF) were increased in PBS-treated TNBS mice and were reduced in TNBS mice treated with *B. dentium* and γ-glutamylcysteine. We also observed decreases in ER stress-related genes GRP-78, CHOP and xsBP1 in *B. dentium*-treated TNBS mice compared with PBS-treated TNBS mice (Figure 5d-F). Furthermore, we noted decreased levels of GRP-78 and CHOP in γ-glutamylcysteine-treated TNBS mice compared with PBS-treated TNBS mice.

**Figure 6.**
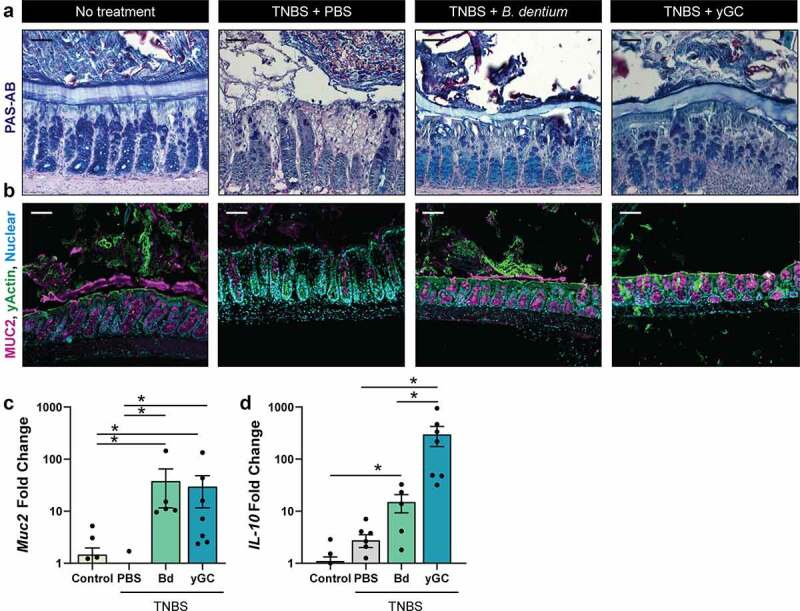
***B. dentium* and γ-glutamylcysteine promote the retention of colonic goblet cells and mucus. a**. Representative images of PAS-AB stains of untreated control animals and TNBS treated animals receiving PBS vehicle, live *B. dentium* or γ-glutamylcysteine, and *B. dentium* mono-associated colon (20x, scale bar = 50 µm). **b**. Representative images of MUC2 and γ-actin of untreated control animals and TNBS-treated animals receiving PBS vehicle, live *B. dentium* or γ-glutamylcysteine, and *B. dentium* mono-associated colon (scale bar = 50 µm). **c**. Colonic mRNA expression of Muc2 in untreated control animals or TNBS-treated animals receiving PBS-vehicle, *B. dentium* (Bd) or γ-glutamylcysteine (yGC). **d**. Colonic mRNA expression of IL-10 in untreated control animals or TNBS-treated animals receiving PBS-vehicle, *B. dentium* (Bd) or γ-glutamylcysteine (yGC). n = 5 mice/group. *p < .05, One-Way ANOVA

Since we observed dramatically enhanced goblet cell numbers in *B. dentium-* and γ-glutamylcysteine-treated TNBS mice, we also assessed goblet cells by PAS-AB and immunostaining (Figure 6a,b). While the mucus layer was disrupted in PBS-treated TNBS mice, *B. dentium* and γ-glutamylcysteine administration promoted retention of the mucus layer and preservation of MUC2-positive goblet cells. Analysis of colonic tissue by qPCR confirmed that *B. dentium* and γ-glutamylcysteine elevated MUC2 and IL-10 levels compared to PBS-treated TNBS mice (Figure 6c,d). Collectively these data support the role of *B. dentium* secreted compounds, such as γ-glutamylcysteine, in promoting IL-10 and suppressing oxidative and ER stress *in vitro* and *in vivo*. These findings point to the potential for *B. dentium* to be used as a targeted therapeutic for goblet cell-related diseases.

## Discussion

Our data indicate a beneficial role for *B. dentium* in reducing activation of ER stress proteins GRP-78, CHOP, and xsBP1; proteins that are key mediators of ER stress in goblet cells. Our work also suggests that *B. dentium*-secreted products can suppress ER stress-driven ROS, elevate glutathione levels, suppress NF-kB, and diminish pro-inflammatory cytokines. We have identified that *B. dentium* secretes γ-glutamylcysteine, which mirrors the activity of *B. dentium*-conditioned LDM4 in our studies. Using bone marrow-derived dendritic cells, we found that *B. dentium*-conditioned LDM4 can stimulate IL-10 production, an effect we also observed *in vivo* in gnotobiotic and conventionalized mice. We believe that these two systems, γ-glutamylcysteine synthesis and IL-10 elevation, work in synergy to decrease ER stress and ROS, promote goblet cell homeostasis, and maintain the intestinal mucus layer. This study is among the first to link a commensal microbe and its secreted products to modulation of ER stress.

*Bifidobacteria* is known to beneficially modulate the host.^66,82–91^ Although multiple mechanisms are likely involved, modulation of intestinal mucin production and reduction of inflammation are likely key pathways *Bifidobacteria* employ to promote intestinal homeostasis. *Bifidobacteria* can upregulate MUC2 production^67^ and alleviate ER stress.^66^ Goblet cells are particularly sensitive to ER stress^16,24^ and thus modulation of goblet cell ER stress by *Bifidobacteria* may represent a significant pathway for promoting intestinal health. Although no microbial metabolites have been previously identified which suppress ER stress, we reasoned that γ-glutamylcysteine may ameliorate goblet cell ER stress. γ-glutamylcysteine is known to feed into the glutathione pathway and reduce oxidative stress.^33,35,41,44,49,55^ In this study, we found that *B. dentium* secretes γ-glutamylcysteine, which can be converted into the powerful antioxidant glutathione and suppress oxidative stress. Our work indicates that bacterial secreted products harboring γ-glutamylcysteine, as well as purified γ-glutamylcysteine, enter cells and upregulate glutathione levels. Since ER stress activates ROS, we speculate that bacterial γ-glutamylcysteine can suppress the negative consequences of ER stress by acting on ROS. Recent work has suggested that γ-glutamylcysteine alone may likewise serve as an antioxidant.^92^ Thus, it is possible that γ-glutamylcysteine could also act directly by suppressing ROS. By suppressing ROS, we speculate that γ-glutamylcysteine inhibits activation of NF-kB and its initiation of the ER stress regulator, GRP-78. This is consistent with literature, which suggests that activation of GRP-78 requires ROS.^93^ In this way, we reason that our *B. dentium*-secreted y-glutamylcysteine may be modulating ER stress.

Commensal *Lactobacilli* also harbors the GSHA genes to produce γ-glutamylcysteine. Using the Integrated Microbial Genomes (IMG) database (http://img.jgi.doe.gov), we found that *L. plantarum, L. salivarius, L. antri*, and *L. reuteri* genomes contained the gshA gene (glutamate-cysteine ligase). Interestingly, we found an equal number of *Bifidobacteria* genomes, *B. adolescentis, B. bifidum, B. pseudocatenulatum*, and *B. dentium*, harboring the gshA gene. Using LC-MS/MS, we confirmed that *Lactobacilli* could generate γ-glutamylcysteine (data not shown). However, *B. dentium* produced ~4x higher concentrations of γ-glutamylcysteine than our representative lactobacilli. Moreover, *B. dentium* can bind to MUC2, ^67^ potentially increasing the access of *B. dentium* secreted metabolites such as γ-glutamylcysteine to the host epithelium. Not all lactobacilli species can adhere to intestinal mucus, ^94,95^ which may limit the exposure of the epithelium to this beneficial compound. We have previously demonstrated that *B. dentium* lacks the glycosyl hydrolases necessary to degrade mucin^67^ and secretes compounds, including acetate, that increase MUC2 expression.^67^ This makes *B. dentium* ideal for treatment in mucin-depleted states such as that observed in IBD patients. Another potential benefit of using *Bifidobacteria* is that these microbes can be increased in density by common prebiotics such as inulin, plant-based β-glucans, or oligofructose.^96^ As a result, we propose that bifidobacteria generated γ-glutamylcysteine may provide a suitable strategy for elevating epithelial glutathione and suppressing goblet cell ROS and inflammation.

In addition to production of y-glutamylcysteine, we observed that *B. dentium* conditioned LDM4 stimulated IL-10 production in immune cells. We speculate that *B. dentium*-conditioned media harbors other compounds that promote IL-10. *Bifidobacteria* is decorated in exopolysaccharides (EPS), a cell wall component that can be released into the milieu. Purified EPS from *B. longum* W11 stimulates IL-10 from human peripheral blood mononuclear cells (PBMCs).^97^ EPS from *B. longum* BCRC 14634 also stimulated IL-10 production from J77A.1 macrophages.^98^ Therefore, it is possible that EPS from *B. dentium* could be contributing to IL-10 production by dendritic cells and other immune cells. In addition to secreted compounds such as EPS, *B. dentium* metabolites may also contribute to IL-10 production and ER stress reduction. At present these compounds remain unidentified, but we believe future studies should focus on identifying these molecules.

Previous work has shown that IL-10 alleviates ER stress by regulating recruitment of GRP-78 and promotes secretion of mucins from goblet cells.^4,5,9^ *In vivo*, IL-10 administration in *Winnie* mice reduced MUC2 misfolding and inflammation. Additionally, IL-10 was able to reduce tunicamycin induced ER stress in LS174T mucin-producing cells.^4^ Consistent with these findings, we observed that recombinant IL-10 alleviated tunicamycin- and thapsigargin-driven ER-stress in mucin-producing T84 cells. We believe that *B. dentium* stimulation of dendritic cells to produce IL-10 may also alleviate goblet cell ER stress *in vivo* to promote colonic mucus secretion. Although it is difficult to delineate which route is more important for suppressing ER stress (IL-10 vs γ-glutamylcysteine) in our model, we speculate that both work together during TNBS colitis to suppress inflammation and preserve goblet cell numbers and epithelial barrier integrity.

We selected the T84 human colonic adenocarcinoma cell line in our experiments as it is well characterized by anion^99–103^ and mucin secretion.^73,104^ In T84 cells, approximately 10% of the cell population is mucin-secreting cells.^73,104^ This mirrors the approximately 16% goblet cell population in the human distal colon.^105^ Similar to native goblet cells, T84 cells can be stimulated to secrete mucin by a number of secretagogues, including ATP, calcium ionophore A23187, diacylglycerol (DAG), phorbol ester PMA, forskolin, Vasoactive intestinal peptide (VIP), γ-aminobutryic acid (GABA), and prostaglandin E1.^67,73,104,106,107^ Moreover, inhibition of calcium-activated potassium channels with barium chloride (BaCl2), Trimethylamine (TEA), and quinine, as well as inhibition of calcium mobilization by BAPTA and autophagy by 3-methyladenine (3-MA) reduces mucin output significantly.^67,104,106^ In addition to shared pathways, electron microscopy analysis of T84 goblet-like cells reveals structural similarities to colonic goblet cells^73,107^ and T84 cells can respond to bacterial stimuli to synthesize and secrete MUC2.^67^ T84 cells have also been previously used to examine ER stress, ^73–76^ making this model ideal for our analysis. We speculate that our findings with T84 cells likely have many parallels with native tissue. However, additional studies using human colonic tissue or human colonoids (or organoids) would be beneficial in the future.

The intestinal mucus layer is essential to maintain the proper distance between the luminal contents and the host immune system. The importance of this barrier is highlighted by the fact that disruption of the intestinal mucus layer increases inflammation. This has been elegantly demonstrated in several mouse models (*Winnie*, MUC2*^−/-^*, AGR2*^−/-^*, glycan-deficiency, *etc*.).^4,108–112^ Moreover, these animal model phenotypes appear to mirror findings in IBD patients.^31,113–116^ Ulcerative colitis patients in particular have abnormal goblet cell number, altered mucin glycosylation, decreased mucus layer thickness, and reduced mucus integrity.^22,114–119^ Loss of both the thickness and integrity of the mucus layer is thought to promote bacterial-epithelial interactions and drive inflammation.^120,121^ Chronic inflammation leads to ER stress and activation of NF-κB.^18^ The cycle of inflammation and ER stress responses is speculated to worsen IBD.^9,32^

Although antioxidants protect cells from damage induced by ROS, long-term use of antioxidants can increase the risk of some forms of cancer. For example, N-acetylcysteine (NAC), another compound which feeds into the glutathione pathway, increases the risk and accelerates lung cancer progression in mice.^122,123^ These findings suggest that long-term administration of ROS suppressing compounds, such as γ-glutamylcysteine, should be approached with caution. *B. dentium* has a relative abundance of 3.8% according to the Human Microbiome Project consortium and other studies.^67,124–128^ Since *B. dentium* does not make up a large portion of the microbiome under normal conditions, we predict that oral administration of *B. dentium* in patients would likely only elevate *B. dentium* concentrations short term and that *B. dentium levels* would return to baseline after administration had ceased. Further studies are necessary to identify the ability of *B. dentium* to colonize the colon long term in adults.

Given the link between ER stress, mucus production, and inflammation, many researchers and clinicians have begun looking into reducing ER stress as a potential therapeutic target for IBD. Our work points to the novel role of *B. dentium* in alleviating ER stress, promoting mucus production, and minimizing inflammation. *B. dentium* is already a member of the healthy human gut microbiome and could be employed to promote a healthy gut. Based on these findings, we believe that *B. dentium* could serve as a next-generation probiotic for intestinal diseases associated with ER stress and disrupted mucus, such as IBD.

## Supplementary Material

Supplemental Material

## References

[1] Iizuka M. Wound healing of intestinal epithelial cells. World J Gastroenterol. 2011;17(17):2161–21. doi:10.3748/wjg.v17.i17.2161. 21633524 PMC3092866

[2] McGuckin MA, Linden SK, Sutton P, Florin TH. Mucin dynamics and enteric pathogens. Nat Rev Microbiol. 2011;9(4):265–278. doi:10.1038/nrmicro2538. 21407243

[3] Johansson ME, Phillipson M, Petersson J, Velcich A, Holm L, Hansson GC. The inner of the two Muc2 mucin-dependent mucus layers in colon is devoid of bacteria. Proc Natl Acad Sci U S A. 2008;105(39):15064–15069. doi:10.1073/pnas.0803124105. 18806221 PMC2567493

[4] Hasnain SZ, Tauro S, Das I, Tong H, Chen AC, Jeffery PL, McDonald V, Florin TH, McGuckin MA. IL-10 promotes production of intestinal mucus by suppressing protein misfolding and endoplasmic reticulum stress in goblet cells. Gastroenterology. 2013;144(2):357–368 e9. doi:10.1053/j.gastro.2012.10.043. 23123183

[5] Hasnain SZ, Lourie R, Das I, Chen AC, McGuckin MA. The interplay between endoplasmic reticulum stress and inflammation. Immunol Cell Biol. 2012;90(3):260–270. doi:10.1038/icb.2011.112. 22249202 PMC7165805

[6] Chakrabarti A, Chen AW, Varner JD. A review of the mammalian unfolded protein response. Biotechnol Bioeng. 2011;108:2777–2793. 21809331 10.1002/bit.23282 PMC3193940

[7] Walter P, Ron D. The unfolded protein response: from stress pathway to homeostatic regulation. Science. 2011;334(6059):1081–1086. doi:10.1126/science.1209038. 22116877

[8] Pahl HL, Baeuerle PA. Activation of NF-κB by ER stress requires both Ca 2+ and reactive oxygen intermediates as messengers. FEBS Lett. 1996;392(2):129–136. doi:10.1016/0014-5793(96)00800-9. 8772190

[9] Shkoda A, Ruiz PA, Daniel H, Kim SC, Rogler G, Sartor RB, Haller D. Interleukin-10 blocked endoplasmic reticulum stress in intestinal epithelial cells: impact on chronic inflammation. Gastroenterology. 2007;132(1):190–207. doi:10.1053/j.gastro.2006.10.030. 17241871

[10] Malhotra JD, Kaufman RJ. Endoplasmic reticulum stress and oxidative stress: a vicious cycle or a double-edged sword? Antioxid Redox Signal. 2007;9(12):2277–2294. doi:10.1089/ars.2007.1782. 17979528

[11] Tu BP, Weissman JS. Oxidative protein folding in eukaryotes: mechanisms and consequences. J Cell Biol. 2004;164(3):341–346. doi:10.1083/jcb.200311055. 14757749 PMC2172237

[12] Hotamisligil GS. Endoplasmic reticulum stress and the inflammatory basis of metabolic disease. Cell. 2010;140(6):900–917. doi:10.1016/j.cell.2010.02.034. 20303879 PMC2887297

[13] Matus S, Glimcher LH, Hetz C. Protein folding stress in neurodegenerative diseases: a glimpse into the ER. Curr Opin Cell Biol. 2011;23(2):239–252. doi:10.1016/j.ceb.2011.01.003. 21288706

[14] Penke B, Bogar F, Fulop L. Protein Folding and Misfolding, Endoplasmic Reticulum Stress in Neurodegenerative Diseases: in Trace of Novel Drug Targets. Current Protein & Peptide Science. 2016;17(2):169–182. doi:10.2174/1389203716666151102104653. 26521955

[15] Mhaille AN, McQuaid S, Windebank A, Cunnea P, McMahon J, Samali A, FitzGerald U. Increased expression of endoplasmic reticulum stress-related signaling pathway molecules in multiple sclerosis lesions. J Neuropathol Exp Neurol. 2008;67(3):200–211. doi:10.1097/NEN.0b013e318165b239. 18344911

[16] Cao SS. Epithelial ER Stress in Crohnʼs Disease and Ulcerative Colitis. Inflamm Bowel Dis. 2016;22(4):984–993. doi:10.1097/MIB.0000000000000660. 26950312

[17] McGuckin MA, Eri RD, Das I, Lourie R, Florin TH. ER stress and the unfolded protein response in intestinal inflammation. Am J Physiol Gastrointest Liver Physiol. 2010;298(6):G820–32. doi:10.1152/ajpgi.00063.2010. 20338921

[18] Zhang K, Kaufman RJ. From endoplasmic-reticulum stress to the inflammatory response. Nature. 2008;454(7203):455–462. doi:10.1038/nature07203. 18650916 PMC2727659

[19] Hybiske K, Fu Z, Schwarzer C, Tseng J, Do J, Huang N, Machen TE. Effects of cystic fibrosis transmembrane conductance regulator and ΔF508CFTR on inflammatory response, ER stress, and Ca 2+ of airway epithelia. Am J Physiol Lung Cell Mol Physiol. 2007;293(5):L1250–60. doi:10.1152/ajplung.00231.2007. 17827250

[20] Treton X, Pedruzzi E, Cazals–Hatem D, Grodet A, Panis Y, Groyer A, Moreau R, Bouhnik Y, Daniel F, Ogier–Denis E. Altered endoplasmic reticulum stress affects translation in inactive colon tissue from patients with ulcerative colitis. Gastroenterology. 2011;141(3):1024–1035. doi:10.1053/j.gastro.2011.05.033. 21699776

[21] Kaser A, Lee A-H, Franke A, Glickman JN, Zeissig S, Tilg H, Nieuwenhuis EE, Higgins DE, Schreiber S, Glimcher LH, et al. XBP1 links ER stress to intestinal inflammation and confers genetic risk for human inflammatory bowel disease. Cell. 2008;134(5):743–756. doi:10.1016/j.cell.2008.07.021. 18775308 PMC2586148

[22] Heazlewood CK, Cook MC, Eri R, Price GR, Tauro SB, Taupin D, Thornton DJ, Png CW, Crockford TL, Cornall RJ, et al. Aberrant mucin assembly in mice causes endoplasmic reticulum stress and spontaneous inflammation resembling ulcerative colitis. PLoS Med. 2008;5(3):e54. doi:10.1371/journal.pmed.0050054. 18318598 PMC2270292

[23] Bertolotti A, Wang X, Novoa I, Jungreis R, Schlessinger K, Cho JH, West AB, Ron D. Increased sensitivity to dextran sodium sulfate colitis in IRE1β-deficient mice. J Clin Invest. 2001;107(5):585–593. doi:10.1172/JCI11476. 11238559 PMC199427

[24] Cao SS, Zimmermann EM, Chuang BM, Song B, Nwokoye A, Wilkinson JE, Eaton KA, Kaufman RJ. The unfolded protein response and chemical chaperones reduce protein misfolding and colitis in mice. Gastroenterology. 2013;144(5):989–1000 e6. doi:10.1053/j.gastro.2013.01.023. 23336977 PMC3751190

[25] Treton X, Pedruzzi E, Guichard C, Ladeiro Y, Sedghi S, Vallee M, Fernandez N, Bruyere E, Woerther P-L, Ducroc R, et al. Combined NADPH oxidase 1 and interleukin 10 deficiency induces chronic endoplasmic reticulum stress and causes ulcerative colitis-like disease in mice. PLoS One. 2014;9(7):e101669. doi:10.1371/journal.pone.0101669. 25014110 PMC4090121

[26] Hino K, Saito A, Asada R, Kanemoto S, Imaizumi K, Hendershot LM. Increased susceptibility to dextran sulfate sodium-induced colitis in the endoplasmic reticulum stress transducer OASIS deficient mice. PLoS One. 2014;9(2):e88048. doi:10.1371/journal.pone.0088048. 24498426 PMC3912207

[27] Namba T, Tanaka K, Ito Y, Ishihara T, Hoshino T, Gotoh T, Endo M, Sato K, Mizushima T. Positive role of CCAAT/enhancer-binding protein homologous protein, a transcription factor involved in the endoplasmic reticulum stress response in the development of colitis. Am J Pathol. 2009;174(5):1786–1798. doi:10.2353/ajpath.2009.080864. 19359519 PMC2671267

[28] Waldschmitt N, Berger E, Rath E, Sartor RB, Weigmann B, Heikenwalder M, Gerhard M, Janssen K-P, Haller D. C/EBP homologous protein inhibits tissue repair in response to gut injury and is inversely regulated with chronic inflammation. Mucosal Immunol. 2014;7(6):1452–1466. doi:10.1038/mi.2014.34. 24850428

[29] Laukens D, Devisscher L, Van Den Bossche L, Hindryckx P, Vandenbroucke RE, Vandewynckel Y-P, Cuvelier C, Brinkman BM, Libert C, Vandenabeele P, et al. Tauroursodeoxycholic acid inhibits experimental colitis by preventing early intestinal epithelial cell death. Lab Invest. 2014;94(12):1419–1430. doi:10.1038/labinvest.2014.117. 25310532

[30] Brandl K, Rutschmann S, Li X, Du X, Xiao N, Schnabl B, Brenner DA, Beutler B. Enhanced sensitivity to DSS colitis caused by a hypomorphic Mbtps1 mutation disrupting the ATF6-driven unfolded protein response. Proc Natl Acad Sci U S A. 2009;106(9):3300–3305. doi:10.1073/pnas.0813036106. 19202076 PMC2651297

[31] Zhao F, Edwards R, Dizon D, Afrasiabi K, Mastroianni JR, Geyfman M, Ouellette AJ, Andersen B, Lipkin SM. Disruption of Paneth and goblet cell homeostasis and increased endoplasmic reticulum stress in Agr2−/− mice. Dev Biol. 2010;338(2):270–279. doi:10.1016/j.ydbio.2009.12.008. 20025862 PMC2937056

[32] Eri RD, Adams RJ, Tran TV, Tong H, Das I, Roche DK, Oancea I, Png CW, Jeffery PL, Radford-Smith GL, et al. An intestinal epithelial defect conferring ER stress results in inflammation involving both innate and adaptive immunity. Mucosal Immunol. 2011;4(3):354–364. doi:10.1038/mi.2010.74. 21107311 PMC3130192

[33] Crespo I, San-Miguel B, Prause C, Marroni N, Cuevas MJ, Gonzalez-Gallego J, Tunon MJ, Foligne B. Glutamine treatment attenuates endoplasmic reticulum stress and apoptosis in TNBS-induced colitis. PLoS One. 2012;7(11):e50407. doi:10.1371/journal.pone.0050407. 23209735 PMC3508929

[34] Takagi T, Homma T, Fujii J, Shirasawa N, Yoriki H, Hotta Y, Higashimura Y, Mizushima K, Hirai Y, Katada K, et al. Elevated ER stress exacerbates dextran sulfate sodium-induced colitis in PRDX4-knockout mice. Free Radic Biol Med. 2019;134:153–164. doi:10.1016/j.freeradbiomed.2018.12.024. 30578917

[35] Ardite E, Sans M, Panes J, Romero FJ, Pique JM, Fernandez-Checa JC. Replenishment of glutathione levels improves mucosal function in experimental acute colitis. Lab Invest. 2000;80(5):735–744. doi:10.1038/labinvest.3780077. 10830784

[36] Grisham MB, Volkmer C, Tso P, Yamada T. Metabolism of trinitrobenzene sulfonic acid by the rat colon produces reactive oxygen species. Gastroenterology. 1991;101(2):540–547. doi:10.1016/0016-5085(91)90036-K. 1648528

[37] Ali I, Liu HX, Zhong-Shu L, Dong-Xue M, Xu L, Shah SZA, Ullah O, Nan-Zhu F. Reduced glutathione alleviates tunicamycin-induced endoplasmic reticulum stress in mouse preimplantation embryos. J Reprod Dev. 2018;64(1):15–24. doi:10.1262/jrd.2017-055. 29081452 PMC5830354

[38] Wang Q, Hou Y, Yi D, Wang L, Ding B, Chen X, Long M, Liu Y, Wu G. Protective effects of N-acetylcysteine on acetic acid-induced colitis in a porcine model. BMC Gastroenterol. 2013;13(1):133. doi:10.1186/1471-230X-13-133. 24001404 PMC3844587

[39] Seril DN, Liao J, Ho KL, Yang CS, Yang G-Y. Inhibition of chronic ulcerative colitis-associated colorectal adenocarcinoma development in a murine model by N-acetylcysteine. Carcinogenesis. 2002;23(6):993–1001. doi:10.1093/carcin/23.6.993. 12082021

[40] Van Der Vlies D, Makkinje M, Jansens A, Braakman I, Verkleij AJ, Wirtz KW, Post JA. Oxidation of ER resident proteins upon oxidative stress: effects of altering cellular redox/antioxidant status and implications for protein maturation. Antioxid Redox Signal. 2003;5(4):381–387. doi:10.1089/152308603768295113. 13678525

[41] Hwang C, Sinskey AJ, Lodish HF. Oxidized redox state of glutathione in the endoplasmic reticulum. Science. 1992;257(5076):1496–1502. doi:10.1126/science.1523409. 1523409

[42] Malhotra JD, Miao H, Zhang K, Wolfson A, Pennathur S, Pipe SW, Kaufman RJ. Antioxidants reduce endoplasmic reticulum stress and improve protein secretion. Proc Natl Acad Sci U S A. 2008;105(47):18525–18530. doi:10.1073/pnas.0809677105. 19011102 PMC2587584

[43] Chakravarthi S, Bulleid NJ. Glutathione is required to regulate the formation of native disulfide bonds within proteins entering the secretory pathway. J Biol Chem. 2004;279(38):39872–39879. doi:10.1074/jbc.M406912200. 15254031

[44] Chakravarthi S, Jessop CE, Bulleid NJ. The role of glutathione in disulphide bond formation and endoplasmic-reticulum-generated oxidative stress. EMBO reports. 2006;7(3):271–275. doi:10.1038/sj.embor.7400645. 16607396 PMC1456887

[45] Gansemer ER, McCommis KS, Martino M, King-mcalpin AQ, Potthoff MJ, Finck BN, Taylor EB, Rutkowski DT. NADPH and Glutathione Redox Link TCA Cycle Activity to Endoplasmic Reticulum Homeostasis. iScience. 2020;23(5):101116. doi:10.1016/j.isci.2020.101116. 32417402 PMC7254477

[46] Sido B, Hack V, Hochlehnert A, Lipps H, Herfarth C, Droge W. Impairment of intestinal glutathione synthesis in patients with inflammatory bowel disease. Gut. 1998;42(4):485–492. doi:10.1136/gut.42.4.485. 9616308 PMC1727080

[47] Lih-Brody L, Powell SR, Collier KP, Reddy GM, Cerchia R, Kahn E, Weissman GS, Katz S, Floyd RA, McKinley MJ, et al. Increased oxidative stress and decreased antioxidant defenses in mucosa of inflammatory bowel disease. Dig Dis Sci. 1996;41(10):2078–2086. doi:10.1007/BF02093613. 8888724

[48] Buffinton GD, Doe WF. Depleted mucosal antioxidant defences in inflammatory bowel disease. Free Radic Biol Med. 1995;19(6):911–918. doi:10.1016/0891-5849(95)94362-H. 8582668

[49] Holmes EW, Yong SL, Eiznhamer D, Keshavarzian A. Glutathione content of colonic mucosa: evidence for oxidative damage in active ulcerative colitis. Dig Dis Sci. 1998;43(5):1088–1095. doi:10.1023/A:1018899222258. 9590426

[50] Sido B, Lasitschka F, Giese T, Gassler N, Funke B, Schröder–Braunstein J, Brunnemer U, Meuer SC, Autschbach F. A prominent role for mucosal cystine/cysteine metabolism in intestinal immunoregulation. Gastroenterology. 2008;134(1):179–191. doi:10.1053/j.gastro.2007.11.001. 18061179

[51] Moura FA, De Andrade KQ, Jcf DS, Araujo ORP, Goulart MOF. Antioxidant therapy for treatment of inflammatory bowel disease: does it work? Redox Biol. 2015;6:617–639. 26520808 10.1016/j.redox.2015.10.006 PMC4637335

[52] Zhang H, Forman HJ, Choi J. Gamma-glutamyl transpeptidase in glutathione biosynthesis. Methods Enzymol. 2005;401:468–483. 16399403 10.1016/S0076-6879(05)01028-1

[53] Furukawa T, Meydani SN, Blumberg JB. Reversal of age-associated decline in immune responsiveness by dietary glutathione supplementation in mice. Mech Ageing Dev. 1987;38(2):107–117. doi:10.1016/0047-6374(87)90071-6. 3600048

[54] Witschi A, Reddy S, Stofer B, Lauterburg BH. The systemic availability of oral glutathione. Eur J Clin Pharmacol. 1992;43(6):667–669. doi:10.1007/BF02284971. 1362956

[55] Allen J, Bradley RD. Effects of oral glutathione supplementation on systemic oxidative stress biomarkers in human volunteers. J Altern Complement Med. 2011;17(9):827–833. doi:10.1089/acm.2010.0716. 21875351 PMC3162377

[56] Dringen R, Kranich O, Loschmann P-A, Hamprecht B. Use of dipeptides for the synthesis of glutathione by astroglia-rich primary cultures. J Neurochem. 2002;69(2):868–874. doi:10.1046/j.1471-4159.1997.69020868.x. 9231749

[57] Rubio-Aliaga I, Frey I, Boll M, Groneberg DA, Eichinger HM, Balling R, Daniel H. Targeted disruption of the peptide transporter Pept2 gene in mice defines its physiological role in the kidney. Mol Cell Biol. 2003;23(9):3247–3252. doi:10.1128/MCB.23.9.3247-3252.2003. 12697824 PMC153205

[58] Zhou J, Liu W, Pong R-C, Hao G, Sun X, Hsieh J-T. Analysis of oligo-arginine cell-permeable peptides uptake by prostate cells. Amino Acids. 2012;42(4):1253–1260. doi:10.1007/s00726-010-0817-7. 21120551

[59] Chothe P, Singh N, Ganapathy V. Evidence for two different broad-specificity oligopeptide transporters in intestinal cell line Caco-2 and colonic cell line CCD841. Am J Physiol Cell Physiol. 2011;300(6):C1260–9. doi:10.1152/ajpcell.00299.2010. 21307350

[60] Roberts PR, Burney JD, Black KW, Zaloga GP. Effect of chain length on absorption of biologically active peptides from the gastrointestinal tract. Digestion. 1999;60(4):332–337. doi:10.1159/000007679. 10394027

[61] Lochman P, Adam T, Friedecky D, Hlidkova E, Skopkova Z. High-throughput capillary electrophoretic method for determination of total aminothiols in plasma and urine. Electrophoresis. 2003;24(78):1200–1207. doi:10.1002/elps.200390154. 12707912

[62] Richman PG, Meister A. Regulation of gamma-glutamyl-cysteine synthetase by nonallosteric feedback inhibition by glutathione. J Biol Chem. 1975;250(4):1422–1426. doi:10.1016/S0021-9258(19)41830-9. 1112810

[63] Chandler SD, Zarka MH, Vinaya Babu SN, Suhas YS, Raghunatha Reddy KR, Bridge WJ. Safety assessment of gamma-glutamylcysteine sodium salt. Regul Toxicol Pharmacol. 2012;64(1):17–25. doi:10.1016/j.yrtph.2012.05.008. 22698997

[64] Zarka MH, Bridge WJ. Oral administration of γ-glutamylcysteine increases intracellular glutathione levels above homeostasis in a randomised human trial pilot study. Redox Biol. 2017;11:631–636. doi:10.1016/j.redox.2017.01.014. 28131081 PMC5284489

[65] Yang Y, Li L, Hang Q, Fang Y, Dong X, Cao P, Yin Z, Luo L. γ-glutamylcysteine exhibits anti-inflammatory effects by increasing cellular glutathione level. Redox Biol. 2019;20:157–166. doi:10.1016/j.redox.2018.09.019. 30326393 PMC6197438

[66] Akiyama T, Oishi K, Wullaert A, Blachier F. Bifidobacteria Prevent Tunicamycin-Induced Endoplasmic Reticulum Stress and Subsequent Barrier Disruption in Human Intestinal Epithelial Caco-2 Monolayers. PLoS One. 2016;11(9):e0162448. doi:10.1371/journal.pone.0162448. 27611782 PMC5017626

[67] Engevik MA, Luk B, Chang-Graham AL, Hall A, Herrmann B, Ruan W, Endres BT, Shi Z, Garey KW, Hyser JM, et al. Bifidobacterium dentium Fortifies the Intestinal Mucus Layer via Autophagy and Calcium Signaling Pathways. mBio. 2019;10(3). doi:10.1128/mBio.01087-19. PMC6581858 31213556

[68] Yu Y, Ge N, Xie M, Sun W, Burlingame S, Pass AK, Nuchtern JG, Zhang D, Fu S, Schneider MD, et al. Phosphorylation of Thr-178 and Thr-184 in the TAK1 T-loop Is Required for Interleukin (IL)-1-mediated Optimal NFκB and AP-1 Activation as Well as IL-6 Gene Expression. J Biol Chem. 2008;283(36):24497–24505. doi:10.1074/jbc.M802825200. 18617512 PMC2528992

[69] Matheu MP, Sen D, Cahalan MD, Parker I. Generation of bone marrow derived murine dendritic cells for use in 2-photon imaging. J Vis Exp. 2008. doi:10.3791/773. PMC3278326 19066518

[70] Fernando EH, Dicay M, Stahl M, Gordon MH, Vegso A, Baggio C, Alston L, Lopes F, Baker K, Hirota S, et al. A simple, cost-effective method for generating murine colonic 3D enteroids and 2D monolayers for studies of primary epithelial cell function. Am J Physiol Gastrointest Liver Physiol. 2017;313(5):G467–G475. doi:10.1152/ajpgi.00152.2017. 28751424

[71] Chang-Graham AL, Danhof HA, Engevik MA, Tomaro-Duchesneau C, Karandikar UC, Estes MK, Versalovic J, Britton RA, Hyser JM. Human Intestinal Enteroids With Inducible Neurogenin-3 Expression as a Novel Model of Gut Hormone Secretion. Cell Mol Gastroenterol Hepatol. 2019;8(2):209–229. doi:10.1016/j.jcmgh.2019.04.010. 31029854 PMC6664234

[72] Engevik MA, Aihara E, Montrose MH, Shull GE, Hassett DJ, Worrell RT. Loss of NHE3 alters gut microbiota composition and influences Bacteroides thetaiotaomicron growth. Am J Physiol Gastrointest Liver Physiol. 2013;305(10):G697–711. doi:10.1152/ajpgi.00184.2013. 24072680 PMC3840232

[73] McCool DJ, Marcon MA, Forstner JF, Forstner GG. The T84 human colonic adenocarcinoma cell line produces mucin in culture and releases it in response to various secretagogues. Biochem J. 1990;267(2):491–500. doi:10.1042/bj2670491. 2110452 PMC1131316

[74] Banerjee A, Ahmed H, Yang P, Czinn SJ, Blanchard TG. Endoplasmic reticulum stress and IRE-1 signaling cause apoptosis in colon cancer cells in response to andrographolide treatment. Oncotarget. 2106;7(27):41432–41444. doi:10.18632/oncotarget.9180. PMC5173070 27166181

[75] Banerjee A, Banerjee V, Czinn S, Blanchard T. Increased reactive oxygen species levels cause ER stress and cytotoxicity in andrographolide treated colon cancer cells. Oncotarget. 2017;8(16):26142–26153. doi:10.18632/oncotarget.15393. 28412728 PMC5432246

[76] Bourgine J, Billaut-Laden I, Happillon M, Lo-Guidice J-M, Maunoury V, Imbenotte M, Broly F. Gene expression profiling of systems involved in the metabolism and the disposition of xenobiotics: comparison between human intestinal biopsy samples and colon cell lines. Drug Metab Dispos. 2012;40(4):694–705. doi:10.1124/dmd.111.042465. 22217464

[77] Dokka S, Shi X, Leonard S, Wang L, Castranova V, Rojanasakul Y. Interleukin-10-mediated inhibition of free radical generation in macrophages. Am J Physiol Lung Cell Mol Physiol. 2001;280(6):L1196–202. doi:10.1152/ajplung.2001.280.6.L1196. 11350798

[78] Latorre E, Matheus N, Layunta E, Alcalde AI, Mesonero JE. IL-10 counteracts proinflammatory mediator evoked oxidative stress in Caco-2 cells. Mediators Inflamm. 2014;2014:982639. doi:10.1155/2014/982639. 25147442 PMC4132333

[79] Rapp UK, Kaufmann SH. Glucose-regulated stress proteins and antibacterial immunity. Trends Microbiol. 2003;11(11):519–526. doi:10.1016/j.tim.2003.09.001. 14607069

[80] Lee AS. The glucose-regulated proteins: stress induction and clinical applications. Trends Biochem Sci. 2001;26(8):504–510. doi:10.1016/S0968-0004(01)01908-9. 11504627

[81] Schulke S. Induction of Interleukin-10 Producing Dendritic Cells As a Tool to Suppress Allergen-Specific T Helper 2 Responses. Front Immunol. 2018;9:455. doi:10.3389/fimmu.2018.00455. 29616018 PMC5867300

[82] Gad M, Ravn P, Soborg DA, Lund-Jensen K, Ouwehand AC, Jensen SS. Regulation of the IL-10/IL-12 axis in human dendritic cells with probiotic bacteria. FEMS Immunol Med Microbiol. 2011;63(1):93–107. doi:10.1111/j.1574-695X.2011.00835.x. 21707779

[83] Jasberg H, Soderling E, Endo A, Beighton D, Haukioja A. Bifidobacteria inhibit the growth of Porphyromonas gingivalis but not of Streptococcus mutans in an in vitro biofilm model. Eur J Oral Sci. 2016;124(3):251–258. doi:10.1111/eos.12266. 27061393

[84] Kirmiz N, Robinson RC, Shah IM, Barile D, Mills DA. Milk Glycans and Their Interaction with the Infant-Gut Microbiota. Annu Rev Food Sci Technol. 2018;9(1):429–450. doi:10.1146/annurev-food-030216-030207. 29580136 PMC5999319

[85] Luk B, Veeraragavan S, Engevik M, Balderas M, Major A, Runge J, Luna RA, Versalovic J, Taneja V. Postnatal colonization with human “infant-type” Bifidobacterium species alters behavior of adult gnotobiotic mice. PLoS One. 2018;13(5):e0196510. doi:10.1371/journal.pone.0196510. 29763437 PMC5953436

[86] Mortensen B, Murphy C, O’Grady J, Lucey M, Elsafi G, Barry L, Westphal V, Wellejus A, Lukjancenko O, Eklund AC, et al. Bifidobacterium breve Bif195 Protects Against Small-Intestinal Damage Caused by Acetylsalicylic Acid in Healthy Volunteers. Gastroenterology. 2019;157(3):637–646 e4. doi:10.1053/j.gastro.2019.05.008. 31095949

[87] Nagpal R, Kurakawa T, Tsuji H, Takahashi T, Kawashima K, Nagata S, Nomoto K, Yamashiro Y. Evolution of gut Bifidobacterium population in healthy Japanese infants over the first three years of life: a quantitative assessment. Sci Rep. 2017;7(1):10097. doi:10.1038/s41598-017-10711-5. 28855672 PMC5577255

[88] O’Callaghan A, Van Sinderen D. Bifidobacteria and Their Role as Members of the Human Gut Microbiota. Front Microbiol. 2016;7:925. doi:10.3389/fmicb.2016.00925. 27379055 PMC4908950

[89] Li N, Yu Y, Chen X, Gao S, Zhang Q, Xu C. Bifidobacterium breve M-16V alters the gut microbiota to alleviate OVA-induced food allergy through IL-33/ST2 signal pathway. J Cell Physiol. 2020. doi:10.1002/jcp.29751. 32394447

[90] Chen Y, Jin Y, Stanton C, Paul Ross R, Zhao J, Zhang H, Yang B, Chen W. Alleviation effects of Bifidobacterium breve on DSS-induced colitis depends on intestinal tract barrier maintenance and gut microbiota modulation. Eur J Nutr. 2020. doi:10.1007/s00394-020-02252-x. 32350653

[91] Hrdy J, Alard J, Couturier-Maillard A, Boulard O, Boutillier D, Delacre M, Lapadatescu C, Cesaro A, Blanc P, Pot B, et al. Lactobacillus reuteri 5454 and Bifidobacterium animalis ssp. lactis 5764 improve colitis while differentially impacting dendritic cells maturation and antimicrobial responses. Sci Rep. 2020;10(1):5345. doi:10.1038/s41598-020-62161-1. 32210304 PMC7093418

[92] Quintana-Cabrera R, Fernandez-Fernandez S, Bobo-Jimenez V, Escobar J, Sastre J, Almeida A, Bolanos JP. γ-Glutamylcysteine detoxifies reactive oxygen species by acting as glutathione peroxidase-1 cofactor. Nat Commun. 2012;3(1):718. doi:10.1038/ncomms1722. 22395609 PMC3316877

[93] Lai M-T, Huang K-L, Chang W-M, Lai Y-K. Geldanamycin induction of grp78 requires activation of reactive oxygen species via ER stress responsive elements in 9L rat brain tumour cells. Cell Signal. 2003;15(6):585–595. doi:10.1016/S0898-6568(03)00004-4. 12681446

[94] Jensen H, Roos S, Jonsson H, Rud I, Grimmer S, Van Pijkeren J-P, Britton RA, Axelsson L. Role of Lactobacillus reuteri cell and mucus-binding protein A (CmbA) in adhesion to intestinal epithelial cells and mucus in vitro. Microbiology (Reading). 2014;160(4):671–681. doi:10.1099/mic.0.073551-0. 24473252 PMC7336543

[95] MacKenzie DA, Jeffers F, Parker ML, Vibert-Vallet A, Bongaerts RJ, Roos S, Walter J, Juge N. Strain-specific diversity of mucus-binding proteins in the adhesion and aggregation properties of Lactobacillus reuteri. Microbiology (Reading). 2010;156(11):3368–3378. doi:10.1099/mic.0.043265-0. 20847011

[96] Meyer D, Stasse-Wolthuis M. The bifidogenic effect of inulin and oligofructose and its consequences for gut health. Eur J Clin Nutr. 2009;63(11):1277–1289. doi:10.1038/ejcn.2009.64. 19690573

[97] Inturri R, Mangano K, Santagati M, Intrieri M, Di Marco R, Blandino G. Immunomodulatory Effects of Bifidobacterium longum W11 Produced Exopolysaccharide on Cytokine Production. Curr Pharm Biotechnol. 2018;18(11):883–889. doi:10.2174/1389201019666171226151551. 29278212

[98] Wu M-H, Pan T-M, Wu Y-J, Chang S-J, Chang M-S, Hu C-Y. Exopolysaccharide activities from probiotic bifidobacterium: immunomodulatory effects (on J774A.1 macrophages) and antimicrobial properties. Int J Food Microbiol. 2010;144(1):104–110. doi:10.1016/j.ijfoodmicro.2010.09.003. 20884069

[99] McRoberts JA, Beuerlein G, Dharmsathaphorn K. Cyclic AMP and Ca2+-activated K+ transport in a human colonic epithelial cell line. J Biol Chem. 1985;260(26):14163–14172. doi:10.1016/S0021-9258(17)38698-2. 2997198

[100] Mandel KG, McRoberts JA, Beuerlein G, Foster ES, Dharmsathaphorn K. Ba2+ inhibition of VIP- and A23187-stimulated Cl- secretion by T84 cell monolayers. Am J Physiol. 1986;250(3):C486–94. doi:10.1152/ajpcell.1986.250.3.C486. 2420200

[101] Mandel KG, Dharmsathaphorn K, McRoberts JA. Characterization of a cyclic AMP-activated Cl-transport pathway in the apical membrane of a human colonic epithelial cell line. J Biol Chem. 1986;261(2):704–712. doi:10.1016/S0021-9258(17)36150-1. 3001077

[102] Cartwright CA, McRoberts JA, Mandel KG, Dharmsathaphorn K. Synergistic action of cyclic adenosine monophosphate- and calcium-mediated chloride secretion in a colonic epithelial cell line. J Clin Invest. 1985;76(5):1837–1842. doi:10.1172/JCI112176. 2997291 PMC424220

[103] Dharmsathaphorn K, Pandol SJ. Mechanism of chloride secretion induced by carbachol in a colonic epithelial cell line. J Clin Invest. 1986;77(2):348–354. doi:10.1172/JCI112311. 3003156 PMC423353

[104] Marcon MA, McCool D, Forstner J, Forstner G. Inhibition of mucin secretion in a colonic adenocarcinoma cell line by DIDS and potassium channel blockers. Biochim Biophys Acta. 1990;1052(1):17–23. doi:10.1016/0167-4889(90)90051-E. 2108728

[105] Karam SM. Lineage commitment and maturation of epithelial cells in the gut. Front Biosci. 1999;4(4):D286–98. doi:10.2741/A426. 10077541

[106] Forstner G, Zhang Y, McCool D, Forstner J. Regulation of mucin secretion in T84 adenocarcinoma cells by forskolin: relationship to Ca2+ and PKC. Am J Physiol. 1994;266(4 Pt 1):G606–12. doi:10.1152/ajpgi.1994.266.4.G606. 8178999

[107] Bradbury NA. Protein kinase-A-mediated secretion of mucin from human colonic epithelial cells. J Cell Physiol. 2000;185(3):408–415. doi:10.1002/1097-4652(200012)185:3<408::AID-JCP11>3.0.CO;2-2. 11056011

[108] Van Der Sluis M, De Koning BA, De Bruijn AC, Velcich A, Meijerink JP, Van Goudoever JB, Buller HA, Dekker J, Van Seuningen I, Renes IB, et al. Muc2-deficient mice spontaneously develop colitis, indicating that MUC2 is critical for colonic protection. Gastroenterology. 2006;131(1):117–129. doi:10.1053/j.gastro.2006.04.020. 16831596

[109] Tadesse S, Corner G, Dhima E, Houston M, Guha C, Augenlicht L, Velcich A. MUC2 mucin deficiency alters inflammatory and metabolic pathways in the mouse intestinal mucosa. Oncotarget. 2017;8(42):71456–71470. doi:10.18632/oncotarget.16886. 29069719 PMC5641062

[110] Bergstrom K, Fu J, Johansson ME, Liu X, Gao N, Wu Q, Song J, McDaniel JM, McGee S, Chen W, et al. Core 1– and 3–derived O-glycans collectively maintain the colonic mucus barrier and protect against spontaneous colitis in mice. Mucosal Immunol. 2017;10(1):91–103. doi:10.1038/mi.2016.45. 27143302 PMC5097036

[111] Maurel M, Obacz J, Avril T, Ding Y-P, Papadodima O, Treton X, Daniel F, Pilalis E, Horberg J, Hou W, et al. Control of anterior GRadient 2 (AGR2) dimerization links endoplasmic reticulum proteostasis to inflammation. EMBO Mol Med. 2019;11(6):11. doi:10.15252/emmm.201810120. PMC6554669 31040128

[112] Park S-W, Zhen G, Verhaeghe C, Nakagami Y, Nguyenvu LT, Barczak AJ, Killeen N, Erle DJ. The protein disulfide isomerase AGR2 is essential for production of intestinal mucus. Proc Natl Acad Sci U S A. 2009;106(17):6950–6955. doi:10.1073/pnas.0808722106. 19359471 PMC2678445

[113] Trabucchi E, Mukenge S, Baratti C, Colombo R, Fregoni F, Montorsi W. Differential diagnosis of Crohn’s disease of the colon from ulcerative colitis: ultrastructure study with the scanning electron microscope. Int J Tissue React. 1986;8:79–84. 3949447

[114] Hanski C, Born M, Foss HD, Marowski B, Mansmann U, Arast�h K, Bachler B, Papenfu� M, Niedobitek F. Defective post-transcriptional processing of MUC2 mucin in ulcerative colitis and in Crohn’s disease increases detectability of the MUC2 protein core. J Pathol. 1999;188(3):304–311. doi:10.1002/(SICI)1096-9896(199907)188:3<304::AID-PATH375>3.0.CO;2-A. 10419600

[115] Wenzel UA, Magnusson MK, Rydstrom A, Jonstrand C, Hengst J, Johansson ME, Velcich A, Ohman L, Strid H, Sjovall H, et al. Spontaneous colitis in Muc2-deficient mice reflects clinical and cellular features of active ulcerative colitis. PLoS One. 2014;9(6):e100217. doi:10.1371/journal.pone.0100217. 24945909 PMC4063762

[116] Tytgat KM, Van Der Wal JW, Einerhand AW, Buller HA, Dekker J. Quantitative analysis of MUC2 synthesis in ulcerative colitis. Biochem Biophys Res Commun. 1996;224(2):397–405. doi:10.1006/bbrc.1996.1039. 8702401

[117] Pullan RD, Thomas GA, Rhodes M, Newcombe RG, Williams GT, Allen A, Rhodes J. Thickness of adherent mucus gel on colonic mucosa in humans and its relevance to colitis. Gut. 1994;35(3):353–359. doi:10.1136/gut.35.3.353. 8150346 PMC1374589

[118] Raouf AH, Tsai HH, Parker N, Hoffman J, Walker RJ, Rhodes JM. Sulphation of colonic and rectal mucin in inflammatory bowel disease: reduced sulphation of rectal mucus in ulcerative colitis. Clin Sci (Lond). 1992;83(5):623–626. doi:10.1042/cs0830623. 1335401

[119] Larsson JM, Karlsson H, Crespo JG, Johansson ME, Eklund L, Sjovall H, Hansson GC. Altered O-glycosylation profile of MUC2 mucin occurs in active ulcerative colitis and is associated with increased inflammation. Inflamm Bowel Dis. 2011;17(11):2299–2307. doi:10.1002/ibd.21625. 21290483

[120] Antoni L. Intestinal barrier in inflammatory bowel disease. World J Gastroenterol. 2014;20(5):1165–1179. doi:10.3748/wjg.v20.i5.1165. 24574793 PMC3921501

[121] Johansson ME, Gustafsson JK, Holmen-Larsson J, Jabbar KS, Xia L, Xu H, Ghishan FK, Carvalho FA, Gewirtz AT, Sjovall H, et al. Bacteria penetrate the normally impenetrable inner colon mucus layer in both murine colitis models and patients with ulcerative colitis. Gut. 2014;63(2):281–291. doi:10.1136/gutjnl-2012-303207. 23426893 PMC3740207

[122] Sayin VI, Ibrahim MX, Larsson E, Nilsson JA, Lindahl P, Bergo MO. Antioxidants accelerate lung cancer progression in mice. Sci Transl Med. 2014;6(221):221ra15. doi:10.1126/scitranslmed.3007653. 24477002

[123] Breau M, Bernard D, Mechta-Grigoriou F, Adnot S. Le traitement antioxydant protège contre l’emphysème mais favorise la survenue et la progression du cancer du poumon. Med Sci (Paris). 2020;36(3):200–203. doi:10.1051/medsci/2020030. 32228832

[124] Human Microbiome PC. Structure, function and diversity of the healthy human microbiome. Nature. 2012;486:207–214. 22699609 10.1038/nature11234 PMC3564958

[125] Pokusaeva K, Johnson C, Luk B, Uribe G, Fu Y, Oezguen N, Matsunami RK, Lugo M, Major A, Mori-Akiyama Y, et al. GABA-producing Bifidobacterium dentium modulates visceral sensitivity in the intestine. Neurogastroenterol Motil. 2017;29(1):e12904. doi:10.1111/nmo.12904. PMC5195897 27458085

[126] Nebra Y, Bonjoch X, Blanch AR. Use of Bifidobacterium dentium as an indicator of the origin of fecal water pollution. Appl Environ Microbiol. 2003;69(5):2651–2656. doi:10.1128/AEM.69.5.2651-2656.2003. 12732533 PMC154539

[127] Ménard O, Butel M-J, Gaboriau-Routhiau V, Waligora-Dupriet A-J. Gnotobiotic mouse immune response induced by Bifidobacterium sp. strains isolated from infants. Appl Environ Microbiol. 2008;74(3):660–666. doi:10.1128/AEM.01261-07. 18083875 PMC2227707

[128] Ventura M, Elli M, Reniero R, Zink R. Molecular microbial analysis of Bifidobacterium isolates from different environments by the species-specific amplified ribosomal DNA restriction analysis (ARDRA). FEMS Microbiol Ecol. 2001;36(2–3):113–121. doi:10.1111/j.1574-6941.2001.tb00831.x. 11451515

