# Supplementary material for: Bifidobacterium dentium-derived y-glutamylcysteine suppresses ER-mediated goblet cell stress and reduces TNBS-driven colonic inflammation
Source: Gut Microbes. 2021 May 14;13(1):1902717. doi: 10.1080/19490976.2021.1902717 (PMC8128206; doi:10.1080/19490976.2021.1902717)
Supplement: Supplemental Material [file KGMI_A_1902717_SM2142.docx]

**Supplemental Methods:**

# Mass Spectrometric Analysis of γ-glutamylcysteine

*Chemicals, Reagents, and Durable Supplies*

The authentic reference material and stable-isotope labeled internal standard (IS) including (Des-Gly)-Glutathione (reduced) trifluoroacetate salt (γ-Glu-Cys) and (Des-Gly, [^13^C_5_, ^15^N_1_])-Glutathione (reduced) trifluoroacetate salt ([^13^C_5_, ^15^N_1_]-γ-Glu-Cys) were from Bachem. Optima™ LC/MS-grade water, acetonitrile (ACN), methanol, and formic acid (FA) were obtained from Fisher Scientific (Waltham, MA, USA) – Optima LC/MS-grade solvents were used throughout the method. HPLC-grade Dithiothreitol (DTT) was purchased from Millipore-Sigma (Burlington, MA, USA).

*Key solutions and Reagents*

A DTT stock solution was prepared at a concentration of 1 M in water. This solution was vortex-mixed for two min to ensure solubility, and was then sub-aliquoted at 100 µL volumes in 0.6 mL polypropylene (PP) microfuge tubes, and stored frozen at -20°C when not in use. A DTT working solution was freshly prepared each day from a DTT stock at a concentration of 5 mM in water. The DTT working solution was vortex-mixed briefly prior to use, and was discarded after use.

A γ-Glu-Cys stock solution was prepared at a concentration of 10 mg/mL in a volume of the 5 mM DTT working solution, and was vortex-mixed for two min to solubilize the material. A γ-Glu-Cys intermediate solution was prepared from the stock solution at a concentration of 100 µg/mL in 5 mM DTT working solution, and was vortex-mixed briefly. Calibration Standards (Calibrators) were prepared from the intermediate solution by serial dilution (DF = 4-fold per Calibrator level) at the following concentrations: 1,000, 250, 62.5, 15.6, 3.90, and 0.977 ng/mL using the 5 mM DTT working solution as the diluent. Calibrators were prepared fresh for each analytical batch, and the γ-Glu-Cys stock and intermediate solutions were sub-aliquotted at 100 µL volumes, and stored frozen at -80°C when not in use. The unused portion of the γ-Glu-Cys stock and intermediate solutions were discarded after use.

A [^13^C_5_, ^15^N_1_]-γ-Glu-Cys IS stock solution was prepared at a concentration of 10 mg/mL in a volume of the 5 mM DTT working solution, and was vortex-mixed for two min to solubilize the material. A [^13^C_5_, ^15^N_1_]-γ-Glu-Cys Working IS (WIS) solution was prepared at a concentration of 10.0 µg/mL in the 5 mM DTT working solution, and was vortex-mixed briefly prior to use. The [^13^C_5_, ^15^N_1_]-γ-Glu-Cys WIS solution was prepared fresh for each analytical batch, and the [^13^C_5_, ^15^N_1_]-γ-Glu-Cys stock solution was sub-aliquotted at 100 µL volumes, and stored frozen at -80°C when not in use. The unused portion of the [^13^C_5_, ^15^N_1_]-γ-Glu-Cys stock and intermediate solutions were discarded after use.

*Calibrator, Blank, and Unknown Sample Preparations*

In 1.5 mL PP microfuge tubes, a 100 µL volume of each Calibrator, blank control media, and filter-sterilized spent media sample was mixed with a 5 µL volume of the [^13^C_5_, ^15^N_1_]-γ-Glu-Cys WIS solution. Blank samples were prepared by mixing a 100 µL volume of PBS with a 5 µL volume of blank 5 mM DTT working solution. All samples were vortex-mixed for 15 seconds, transferred to tapered PP autosampler vials, and a 10 µL volume was injected for analysis on the LC-MS/MS system.

*LC-MS/MS Conditions and Parameters for the Targeted γ-Glu-Cys Method*

The LC-MS/MS method uses reverse-phase chromatography for the quantification of the γ-Glu-Cys content of the LDM4 medium. Chemical separations were performed using an aqueous phase A and organic phase B solution of 0.1% formic acid (FA) in water and 0.1% FA in acetonitrile, respectively. The needle wash solution was comprised of a solution of methanol:water (1:1, *v:v*). Chromatographic separations were performed using a Phenomenex 5-micron Luna amino analytical column (50 x 1.0 mm). The chromatographic method included column heating at 40 °C, autosampler tray cooled to 12°C, a mobile phase flowrate of 0.150 mL/min, and a gradient elution program specified as follows: 0-2 min, 1% B; linearly increased to 80% B over 5 min, held at 80% B for 1 min; dropped back to 1% B over 1 min; held for 2 min at 1% B, yielding a total gradient time of 10 min.

The TurboIonSpray® electrospray ionization (ESI) probe was installed in the Turbo V™ ion source, and was operated with the following source conditions: ionization mode polarity: positive; curtain gas (Cur): 20; TurboIonSpray™ voltage (IS): +5,100 V; source temperature (TEM): 300 °C; Ion Source Gas 1 (GS1; nebulization gas): 30 psi; Ion Source Gas 2 (GS2; heater gas): 30 psi. MS/MS operational parameters include the following: collisionally activated dissociation (CAD) gas pressure: “High”; declustering potential (DP): 60 V; entrance potential: 5 V; collision-cell exit potential (CXP): 9 V; mass analyzer quadrupole 1 (Q1) resolution: unit; Q3 resolution: unit. Selected reaction monitoring (SRM) transitions and corresponding collision energies for each compound were as follows: γ-Glu-Cys: *m/z* 251.1 > *m/z* 84.0 (36 eV), *m/z* 251.1 > *m/z* 130.1 (21 eV), *m/z* 251.1 > *m/z* 188.1 (21 eV); [^13^C_5_, ^15^N_1_]-γ-Glu-Cys: *m/z* 257.2 > *m/z* 89.0 (38 eV), *m/z* 257.2 > *m/z* 122.0 (17 eV), *m/z* 257.2 > *m/z* 136.1 (21 eV).

**Tissue Culture**

*Culturing conditions*

Human colon T84 cells were routinely screened for *Mycoplasma* contamination using the Mycoplasma Detection Kit (Lonza, cat# LT07-518). Cells were seeded at 2 x 10^5^ cells/cm^2^ in 24-well tissue culture treated plates (Corning) until the cells reached confluence.

*Viability Analysis*

For cell viability assays, T84 cells were seeded into 96-well plates at 10,000 cells/mL and incubated overnight. The following day, cells were treated with various concentrations of *B. dentium* LDM4 conditioned media, γ-glutamylcysteine, or IL-10 and incubated for 24-48 hrs. For cell viability, cells were incubated with 5 μg/mL propidium iodide (Sigma Aldrich) for 10 min at 37°C and then read on a Synergy H1 plate reader (Biotek) at excitation 535 nm/emission 620 nm.

*Incorporation of fluorescently-labeled cysteine residues*

To examine the uptake of γ-glutamylcysteine into T84 cells, *B. dentium* was grown in complete LDM4 for 6 hrs anaerobically at 37°C, centrifuged at 5,000 x g for 5 min, washed 2x with anaerobic sterile PBS and re-suspended in LDM4 lacking cysteine and glutamate. Bacteria were then incubated for another 12 hrs and centrifuged to remove the bacteria. The resulting supernatant was incubated with 1 mM Fluorescein-5-Maleimide (Cayman # 16383) in 20mM sodium phosphate buffer, 150mM NaCl, pH 7.2 for 2 hours at room temperature. Non-reacted fluorescein was removed by filtration with Amicon Ultra Centrifugal Filter Units (Millipore Sigma).

**RNA isolation and qPCR**

RNA was extracted from mouse colon stored in TRIZOL and RNA isolated according to manufacturer details (ThermoFisher # 15596018). RNA was converted to cDNA using the SensiFAST cDNA synthesis kit (Bioline USA Inc). Quantitative real-time PCR (qPCR) was performed using FastSYBR Green (ThermoFisher) and 10 nM primers designed using PrimerDesign (ThermoFisher) on a QuantStudio 3 qPCR machine (Applied Biosystems). Relative fold change was calculated using the ΔddCT method with the housekeeping gene 18S.

**Intestinal Tissue Staining**

*Immunofluorescence*

Mouse colon was excised and fixed in 10% Carnoys fixative. Fixed tissue was then embedded in paraffin and 7-µm sections were processed for staining. Tissue sections were deparaffinized in a series of histo-clear solution (National Diagnostics, HS-200) followed by dehydration in ethanol. Antigen retrieval was performed in a pressure cooker on high pressure for 15 minutes in Dako citrate buffer (Dako, S1699). Slides were cooled on ice in antigen retrieval solution. Tissue sections were blocked using Dako serum free protein block (Dako, X0909) for 1 hour and 30 minutes at room temperature. The primary antibody, anti-MUC2 (dilution: 1:200, Rabbit anti-MUC2 Santa Cruz Biotech, cat # sc-15334), was diluted in antibody diluent with background reducing components (Dako, S3022). Slides were incubated overnight with the primary antibody solution at 4° C in a humidified chamber. Sections were washed 3 times in PBS. The secondary antibody (Donkey-anti-rabbit-Alexa Fluor 564) and mouse anti-gamma actin conjugated to Alexa Fluor 488 (dilution 1:100, Santa Cruz Biotech, cat# sc-65638) were diluted in antibody diluent (Dako, S0809). The slides were incubated with the secondary antibody and conjugated gamma actin for 1 hour at room temperature. Hoechst was diluted 1:1000 in PBS (Thermo Fisher Scientific, 62249) and slides incubated with Hoechst for 5 minutes at room temperature. Tissue sections were washed 3 times in PBS for 5 minutes each and were mounted with coverslips using ProLong Gold Antifade (Thermo Fisher Scientific, P36934) prior to imaging. Slides were imaged using a Zeiss Axio Imager M2 microscope with an Axiovision digital imaging system.

*H&E and PAS-AB*

To examine the colonic architecture, paraffin-embedded tissue sections were stained with hematoxylin and eosin (H&E). Additionally, sections were stained with Periodic Acid-Schiff/Alcian blue (PAS-AB) to examine goblet cells and mucus production. H&E and PAS-AB sections were imaged on the Nikon Eclipse 90i (Nikon) using a DS-Fi1-U2 camera (Nikon) with a 20x Plan Apo (NA 0.75) differential interference contrast (DIC) objective. Histological scoring was performed on an Olympus BX41 with a DP71 Olympus camera.

**Serum Cytokine Analysis**

Blood samples were collected from cardiac puncture and serum was collected after centrifugation of blood in EDTA-coated tubes according to manufacturer’s protocol (BD Microtainer tubes # 365974). Samples were assayed using a Cytokine Magnetic bead panel (Millipore, cat. #MCYTOMAG-70k) with a MagPix instrument (Luminex Corporation, Austin, TX) by the Functional Genomics and Microbiome Core of the Texas Medical Center Digestive Diseases Center. Raw data were obtained with Luminex xPONENT for MAGPIX, version 4.2 Build 1324 and analyzed with MILLIPLEX Analyst version 5.1.0.0 standard Build 10/27/2012.
